# Pharmacological action of *Angelica sinensis* polysaccharides: a review

**DOI:** 10.3389/fphar.2024.1510976

**Published:** 2025-01-13

**Authors:** Chunzhen Ren, Yali Luo, Xiaojuan Li, Like Ma, Chunling Wang, Xiaodong Zhi, Xinke Zhao, Yingdong Li

**Affiliations:** ^1^ School of Traditional Chinese and Western Medicine, Gansu University of Chinese Medicine, Lanzhou, China; ^2^ Gansu Province Key Laboratory of Chinese Medicine for the Prevention and Treatment of Chronic Diseases, Lanzhou, China; ^3^ Key Clinical Specialty of the National Health Commission of the People’s Republic of China, Key Specialized Cardiovascular Laboratory National Administration of Traditional Chinese Medicine, Lanzhou, China; ^4^ School of Traditional Chinese Medicine, Jiangsu Medical College, Yancheng, China; ^5^ First School of Clinical Medical, Gansu University of Chinese Medicine, Lanzhou, China; ^6^ Cardiovascular clinical medicine center, Affiliated Hospital of Gansu University of Chinese Medicine, Lanzhou, China

**Keywords:** ASP, chemical structure, improving anemia, liver protection, antitumour, immunomodulation

## Abstract

*Angelica sinensis*, a traditional Chinese herbal medicine and food, which has a long history of clinical application, is used to improve health conditions and treat various diseases. *Angelica sinensis* polysaccharides (ASP), the main active component of this traditional Chinese medicine, have multicomponent, multitarget characteristics and very broad pharmacological activities. They play important roles in the treatment of several diseases. In addition, the effect is significant, which may provide a more comprehensive database and theoretical support for applying ASP in the treatment of disease and could be considered a promising candidate for preventing disease. This review summarizes the research progress on the extraction, chemical structure, pharmacological effects, and mechanisms of ASP and its derivatives by reviewing relevant national and international literature and provides comprehensive information and a reliable basis for the exploration of new treatment strategies involving botanical drugs for disease therapy. Literature information was obtained from scientific ethnobotany and ethnomedicine databases (up to September 2024), mainly from the PubMed, Web of Science, and CNKI databases. The literature has explored the extraction, purification, structure, and pharmacological effects of *Angelica sinensis* polysaccharides. The search keywords for such work included “*Angelica sinensis*” or “*Angelica sinensis* polysaccharides,” and “*pharmacological* effects,” “extraction” and “structure.” Multiple studies have shown that ASP has important pharmacological effects, such as antitumor, anemia-improving, anti-inflammatory, antioxidative, immunomodulatory, hepatoprotective, antifibrotic, hypoglycemic, antiradiation, and antiviral effects, the mechanisms of which appear to involve the regulation of inflammation, oxidative stress, and profibrotic signaling pathways. As a natural polysaccharide, ASP has potential applications as a drug. However, further research should be undertaken to clarify the unconfirmed regulatory mechanisms, conduct standard clinical trials, and evaluate the possible side effects. This review establishes a theoretical foundation for future studies on the structure, mechanism, and clinical use of ASP.

## 1 Introduction

**FIGURE 1:**
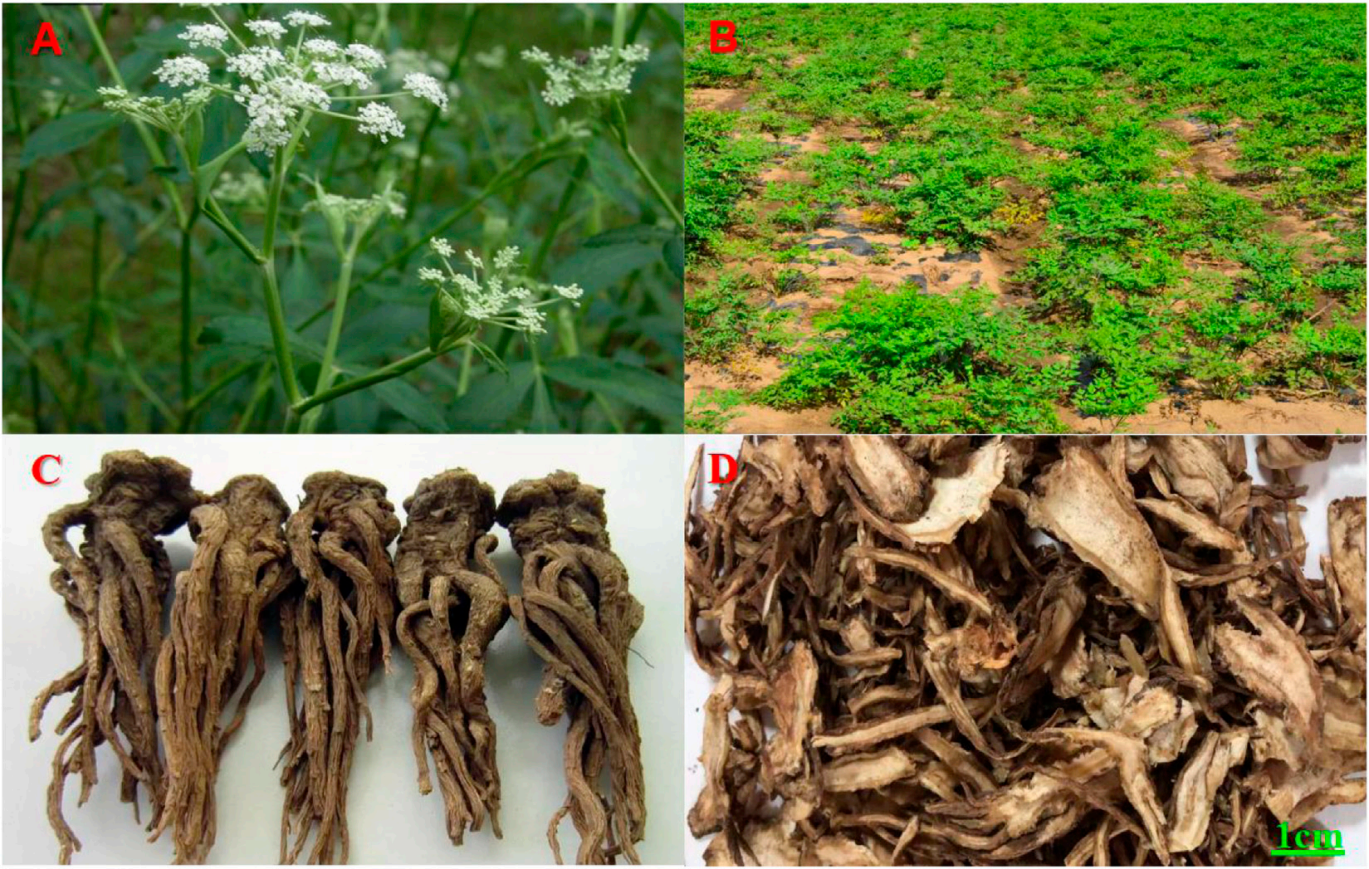
**(A, B)** *Angelica sinensis* herbs (Plant Photo Bank of China, PPBC). **(C)** *Angelica sinensis* original medicinal materialsand (Baidu Gallery). **(D)** *Angelica sinensis* slices (Herbal medicine room, Affiliated Hospital of Gansu University of Chinese Medicine).

*Angelica sinensis* is the dry root of the umbelliferae plant *Angelica sinensis* (Oliv.) Diels, which is recorded in Chinese Pharmacopoeia (2020) (Figures 1A, B). The object exhibits a light brown to brown coloration, characterized by longitudinal wrinkles and transverse lens-like perforations. Its fried slices are typically round, oval, or irregular in shape, displaying a yellow-white to light brown hue with a flat surface that may feature cracks. A light brown cambium ring is present at the center, accompanied by several brown oil spots (Figures 1C, D). *Angelica sinensis* is mainly produced in southeastern Gansu, followed by Yunnan, Sichuan, Shaanxi, Hubei and other provinces. *Angelica sinensis* was first described in Shennong Ben Cao Jing and is known for its ability to tonify blood. It is a traditional medicinal and edible plant that has long been used for invigorating the blood, promoting circulation, lubricating the intestines, regulating menstruation as well as relieving pain, treating female irregular menstruation and amenorrhea (Erlin et al., 2019).

ASP are natural macromolecular compounds and the main active substances of *Angelica sinensis*. They offer a wide range of pharmacological functions, for example, promoting hematopoiesis and improving immunity, antioxidation, and antitumour effects (Jin et al., 2012). It has been widely used owing to its low toxicity, residue-free nature, and nontolerant properties (Jin et al., 2012). As a result, the utilization of ASP is gaining more attention from scholars worldwide. With the deepening of research, the medical potential of ASP is gradually explored. This paper provides an overview of the current research on the extraction, isolation, and pharmacological effects of ASP. Additionally, it analyzes and summarizes the potential value of ASP, thus, serving as a reference for further research on ASP and its application in the fields of food, health products, and pharmaceuticals.

## 2 Methodology

**FIGURE 2:**
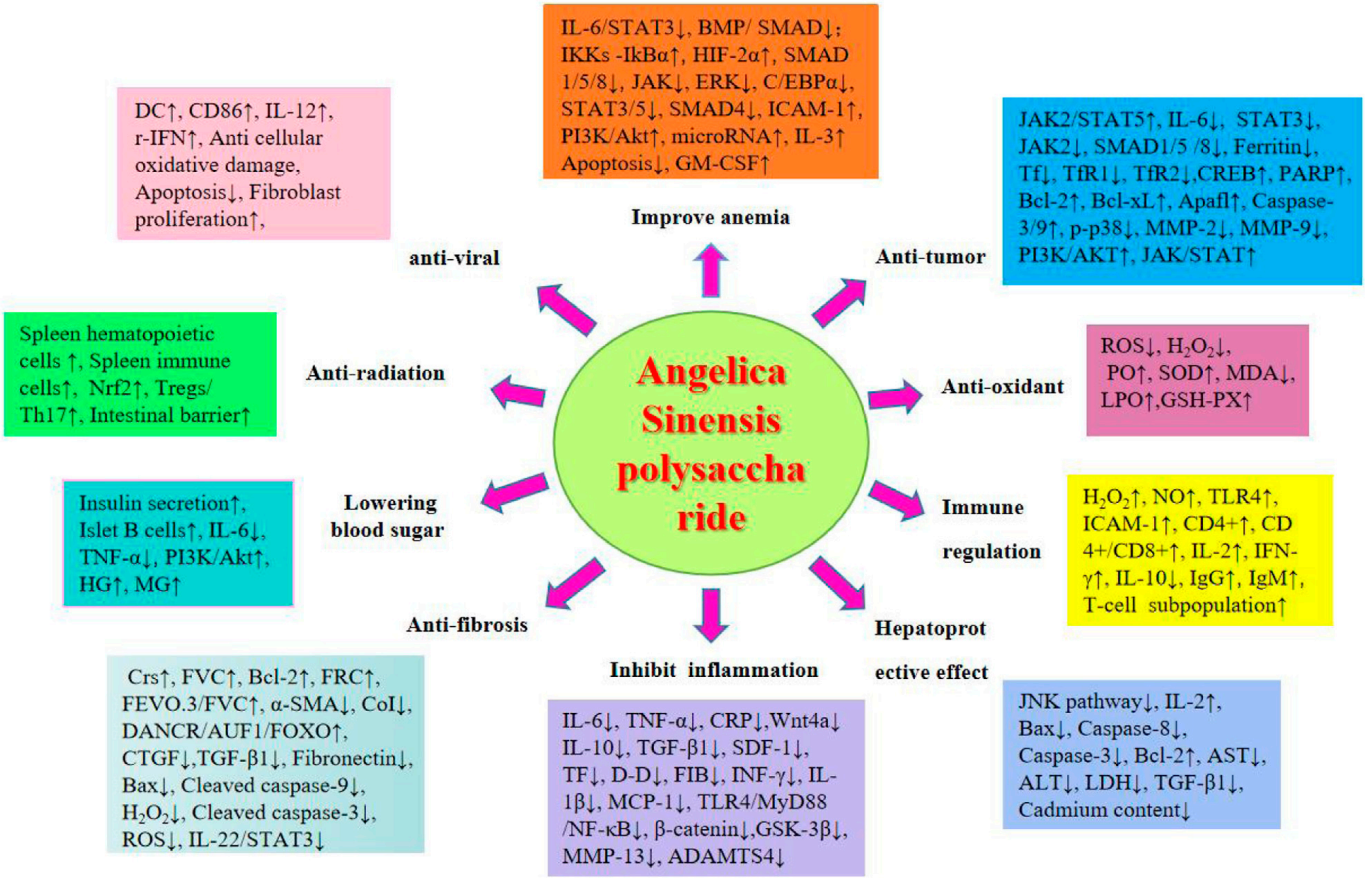
Pharmacological effects of ASP.

This review article has been retrieved in the form of a database search. The search terms are in the form of subject words combined with free words. We systematically searched Baidu Literature, China National Knowledge Infrastructure, VIP Datas, PubMed, Google-Scholar and Web of Science. Using“*Angelica* sinensis”or “*Angelica sinensis* polysaccharides”, and “pharmacological effects”, “extraction” and “structure”, as the search terms from scientific ethnobotany and ethnomedicine databases (up to September 2024), total of 663 articles were retrieved, and 484 were duplicated by software and manual removal. We carefully reviewed and classified the title, abstract and full text of the literature, and finally obtained 21 articles on ASP extraction, separation and structural analysis, 90 pharmacological research studies. The research methods we included include clinical studies, clinical trials, cell experiments, animal experiments, literature reviews, network pharmacology, etc. We extracted study details, including the relevant information on the pharmacological action and chemistry attributes of ASP, as well as the study status. Finally, through literature review and summary, it was found that ASP have ten pharmacological effects, as shown in the following Figure 2.

### 2.1 Extraction of ASP

ASP is a typical traditional Chinese medicinal polysaccharide, and its extraction method is similar to that of other traditional Chinese medicinal polysaccharides. Common extraction methods include water extraction and alcohol precipitation, ultrasound extraction, enzyme extraction, and microwave-assisted extraction.

#### 2.1.1 Water extraction and alcohol precipitation methods

At present, the extraction process of polysaccharides is mostly water extraction and alcohol precipitation, which means that the medicinal material of *Angelica sinensis* is first defatted with organic solvents, then extracted with hot water, and then subjected to alcohol precipitation with a certain concentration of ethanol. The water extraction and alcohol precipitation method has the advantages of simplicity, convenience, low cost, no pollution, and suitability for industrial production. However, a high temperature is required during the extraction process, resulting in a low extraction rate, significant loss of polysaccharide activity, and difficult purification (Huang, 2010). Yu et al. (2013) used response surface methodology and reported that the extraction time and solid‒liquid ratio are the two most important factors affecting the yield of ASP in hot water extraction. Zhang et al. (2016b) soaked *Angelica sinensis* slices in 95% ethanol by volume for 24 h and then extracted them with hot water, followed by alkali and acid precipitation and alcohol precipitation to obtain water-soluble ASP. Wu et al. (2015) adopted an orthogonal design to improve the hot water extraction method of ASP and obtained the optimal extraction process by using 10 cycles of water and 60 min of continuous extraction three times. The obtained aqueous extract was concentrated to 1:8, and 95% ethanol was added to a concentrated solution of 65% to obtain 0.265 mg/g ASP. Zhang et al. (2016a) extracted crude polysaccharides from *Angelica sinensis* using three methods and compared the yield of polysaccharides (fresh *Angelica sinensis* after natural air drying, boiled directly in water; Angelica sinensis was processed into small pieces, soaked in 75% ethanol for 1 week, with a solid‒liquid ratio of 1:10, and the residue after alcohol extraction was boiled in water; *Angelica sinensis* was processed into small pieces, soaked in 78% ethanol for 1 week, with a solid‒liquid ratio of 1:10, and then infiltrated with 76% ethanol until the exudate was colorless. The infiltrated *Angelica sinensis* was boiled in water at a solid‒liquid ratio of 1:30 during decoction and boiled for 30 min. The extraction solutions obtained from the three processing methods were all precipitated with ethanol at a final concentration of 80%, subjected to precipitation and decolorization treatment, and then freeze-dried to obtain crude polysaccharides from *Angelica sinensis*. The results showed that treatment with ethanol could change the yield of crude polysaccharides from *Angelica sinensis*, and the ethanol infiltration method had the highest yield of crude polysaccharides from *Angelica sinensis*.

#### 2.1.2 Ultrasonic extraction method

The ultrasonic extraction method has the advantages of short extraction time, simplicity and speed, and good separation. Zhao et al. (2016) screened the optimal parameters for the ultrasonic extraction of ASP using response surface methodology (a material–liquid ratio of 7, an extraction time of 45 min, an extraction temperature of 90°C, and an ultrasonic power of 180 W). Several scholars (Nai et al., 2021; Tian et al., 2017) have investigated three factors, namely, the material-liquid ratio, sonication time and sonication power, for the extraction of ASP by ultrasound through single-factor tests and orthogonal tests and optimized the extraction solutions by the response surface method. The optimal conditions were a material-liquid ratio of 1:43, an ultrasonication time of 28 min, and a power of 396 W using multiple regression analysis, resulting in a polysaccharide yield of 21%.

#### 2.1.3 Enzyme extraction method

The principle of enzyme extraction is to use enzymes to hydrolyze the cell wall to fully dissolve the substances contained in the cells in water to achieve efficient extraction. Zhang et al. (2012a) used enzyme-assisted extraction technology to extract ASP and optimized the extraction conditions. The results showed that the enzyme extraction method improved the extraction rate of polysaccharides, and the operation was simple, low cost and had little chemical pollution. Zhang and Lv (2013) compared four methods, namely, hot water extraction, ultrasonic extraction, cellulase extraction and pectinase extraction, of ASP, with the yield of ASP as the index. The results showed that the enzymatic method was the better choice for the extraction of ASP. The optimum extraction conditions for the cellulase method were as follows: enzyme dosage, 1.0%; extraction temperature, 60°C; extraction time, 60 min; and pH, 6.0. The optimum extraction conditions for the pectinase method were as follows: enzyme dosage, 1.0%; extraction temperature, 50°C; extraction time, 90 min; and pH, 5.5. Therefore, enzymatic hydrolysis has considerable development prospects.

#### 2.1.4 Microwave-assisted extraction

Microwave-assisted extraction of polysaccharides is a potential new technology that has the advantages of speed, low solvent consumption, high extraction rate and low cost (Zhang, 2006). Jin et al. (2007) studied the extraction of ASP via orthogonal experiments. The results showed that compared with ultrasonic extraction and direct heat extraction, microwave extraction has the advantages of time savings, high efficiency and energy savings. Li et al. (2012) used a single factor grouping test and orthogonal optimization test to investigate the process conditions of microwave-assisted extraction of polysaccharides from *Angelica sinensis*. The results showed that the polysaccharide extraction rate was 7.82% at a power of 500 W, an extraction time of 20 min, and a solid‒liquid ratio of 1:15.

### 2.2 Separation purification of ASP

ASP extracts often contain impurities, such as proteins and pigments, which need to be further separated and purified. Protein impurities are usually removed by the Sevag method (Wang et al., 2019) or the repeated freeze‒thaw method (Yang et al., 2008b). The separation and purification methods used are the common alcohol precipitation method (Wang et al., 2019) and the column chromatography method (Yang et al., 2008b). Among them, the column chromatography methods can be divided into anion exchange chromatography and gel filtration chromatography. Cao et al. (2008b) separated the crude polysaccharide obtained by water extraction and alcohol precipitation and repeated freezing and thawing to remove protein by a DEAE-Sephadex A-25 column and obtained three polysaccharide components. APS-2 was further separated on a Sephacryl S-400 column to obtain two polysaccharide components (APS-2a and APS-2b). Cao et al. (2006) treated ASP with ethanol to remove pigments, extracted it with hot water, and precipitated it with ethanol to obtain crude polysaccharides. The proteins were removed by the freeze‒thaw method, filtered through a 0.65 μm membrane filter, and loaded onto a DEAE Sephadex A-25 column for elution. The eluent was collected, lyophilized, and further passed through a Sephacryl S-400 column to obtain three components (APS-1a, APS-1b, and APS-1c). After concentration, dialysis, and lyophilization, APS-1c was further purified on a SephadexG-100 column to obtain two purified components (APS-1cI and APS-1cII). Zhang et al. (2016b) obtained crude polysaccharides by hot water extraction and ethanoprecipitation. The protein was removed by the freeze‒thaw method, and then, the filtrate was dialyzed and loaded on a Sephadex G-50 column, eluted with distilled water, collected and concentrated, and lyophilized to obtain ASP. With the continuous improvement and development of scientific and effective methods for the preparation of Chinese medicines, approaches for the separation and purification of ASP have been explored.

### 2.3 Structural studies on the ASP

**FIGURE 3:**
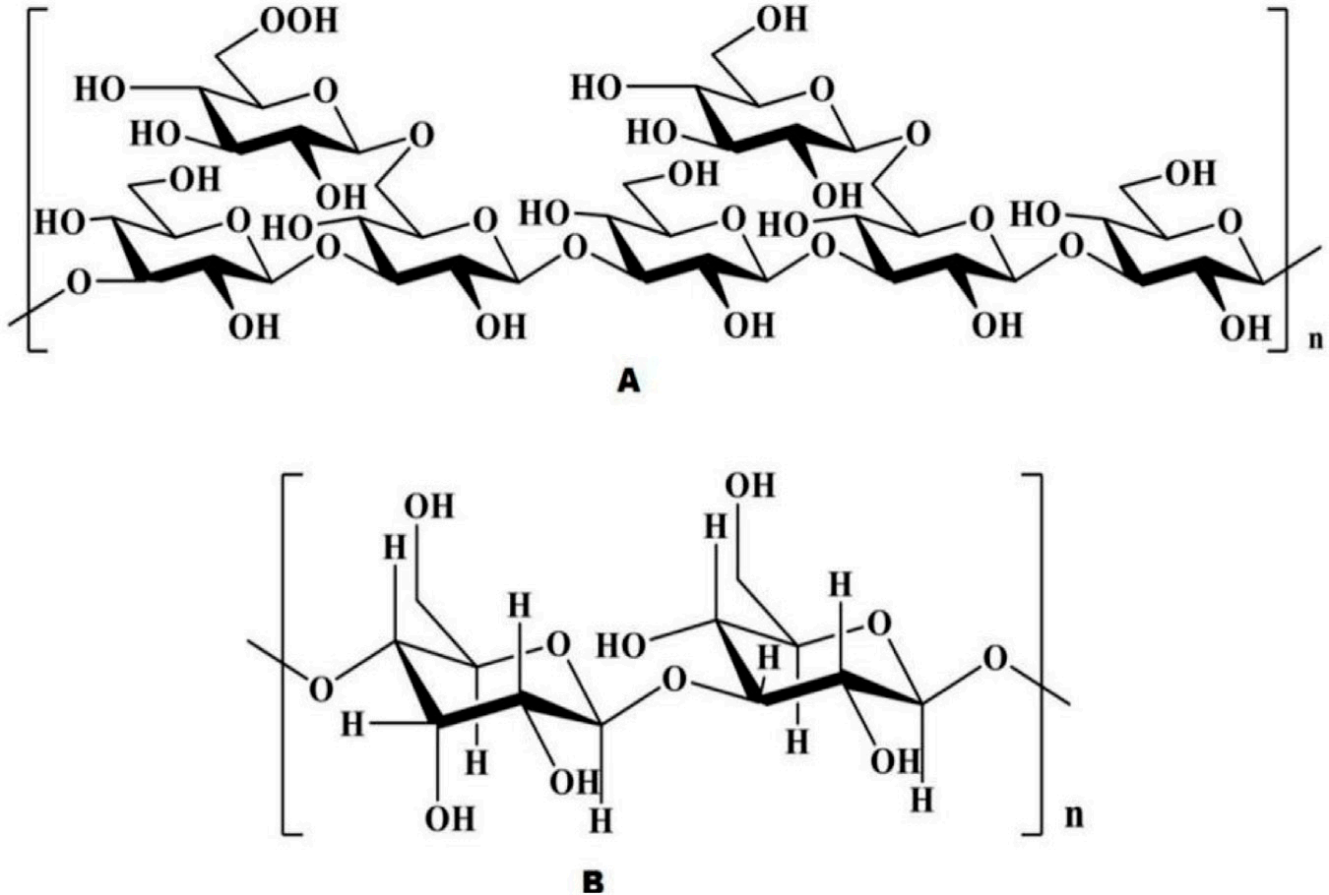
Chemical structure of ASP [Natural Product Communications. 2021; 16(3)].

ASP consists of at least 10 identical or different monosaccharides linked by α- or β-glycosidic bonds and is widely found in nature (Chen and Huang, 2018). Based on their structural characteristics, ASP can be divided into two types: homopolysaccharides, which are composed of one type of monosaccharide, and heteropolysaccharides, which are composed of two or more monosaccharides, such as glucose, galactose, arabinose, and rhamnose, but their components are mainly glucose (Tian et al., 2024). The polysaccharide APS-bII is the first polysaccharide isolated from *Angelica sinensis* in the form of a white powder (Nai et al., 2021). Currently, the polysaccharides extracted from *Angelica sinensis* are mainly heteropolysaccharides (Figure 3), with the main structural backbone being α (1,4)-Glc and a relative molecular weight distribution of 5.1 to 2,300 kDa. The active polysaccharides are mainly water soluble. The physicochemical properties and biological activity of polysaccharides are closely associated with structural parameters such as the monosaccharide backbone, monosaccharide composition, relative molecular mass, conformation, position of glycosidic bonds, functional groups, branching degree, and advanced conformation (Liu et al., 2020a). The polysaccharide species extracted from Angelica are listed in Table 1.

**TABLE 1:** Chemical structure and composition of natural *polysaccharides* from *Angelica sinensis*.

Name	Relative molecular mass (Da)	Monosaccharide composition and molar ratio Structural features	Structural features	Pharmacological activity	References
As-IIIa	8.5 × 10^2^	Glc	α(1→3)-glycosidic bond	Immunomodulation	Zhang and Huang (1999)
As-IIIb	4.9 × 10^4^	Glc-Man-Ara (10.0:10.0:4.0)	(1→4), (1→6) glycosidic bond	Immunomodulation	Zhang and Huang (1999)
X-C-3-II	1.0 × 10^5^	Glc-Gal-Ara-Rha-GalA (56.0:22.1:18.9:1.9:1.1)	—	Immunomodulation	Chen et al. (2001a)
X-C-3-III	8.5 × 10^4^	Gal-Ara-Rha-GlcA-GalA (24.3:15.8:4.2:3.1:52.6)	—	Immunomodulation	Chen et al. (2001b)
X-C-3-IV	6.6 × 10^2^	Gal-Ara-Rha-GleA-GalA (12.6:10.7:7.2 8.3:61.2)Glc	—	Immunomodulation	Chen et al. (2001b)
XC-1	1.0 × 10^5^	Glc	α(1→6)-glycosidic bond	Immunomodulation	Chen et al. (2001c)
ASP1	—	GalA-Ara-Glc-Gal (5.35:9.15:65.00:3.66)	—	Antiradiation	Sun et al. (2005)
ASP2	3.4 × 10*	GalA-Rha-Ara-Man-Glc- Gal (35.38:1.11:16.31:0.89:26.96:15.75)	—	Antiradiation	Sun et al. (2005)
ASP3	8.1 × 10	GalA-Rha-Ara-Man-Gle- Gal (58.27:1.87:10.50:0.37:0.94:24.93)	—	Antiradiation	Sun et al. (2006)
W-ASPll	3.8 × 10^5^	Ara-Glc-Gal (0.5:26.0:0.6)	—	—	Sun et al. (2006)
W-ASP12	1.9 × 10^4^	Ara-Man-Gle-Gal (21.1:1.6:16.3:1.3)	—	—	Sun et al. (2006)
W-ASP-2	—	Rha-Ara-Man-Glc-Ga (1.0:14.7:0.8:24.3:14.2)	—	—	Sun et al. (2006)
W-ASP-3	6.2 × 10^4^	Rha-Ara-Man-Glc-Gal (1.0:5.6:0.2:0.5:13.3)	—	—	Sun et al. (2006)
APS-leI	1.7 × 10^5^	Glc	(1→6)-D-Glcp forming a linear β-glucan	Immunomodulation	Cao et al. (2006)
APS-le II	3.9 × 10^4^	Glc	(1→4)-α-D-Glop and (1→6)-a-DGlep	Antitumor	Cao et al. (2006)
APS-1d	5.1 × 10³	Glc-Ara (13.8:1)	1,4-a-D-glucopyranose with a 1,6-alpha-D-Glcp branched chain	Antitumor	Yang et al. (2006)
AP	5.0 × 10³	Rha-Ara-Man-Glc-Gal (1.00:4.54:2.98:11.09:7.45)	—	Immunomodulation	Wang et al. (2007c)
ASD II-3–3	4.4 × 10^4^	Rha-Ara-Xyl-Man-Gal (0.3:1.0:0.1:0.2:5.0)	—	—	Yang et al. (2008b)
APF1	1.2 × 10^5^	Rha-Ara-Glc-Gal (1.00:2.27:7.80:2.69	—	Immunostimulant	Cao et al. (2008a), Yang et al. (2008a)
APF2	5.2 × 10^4^	Rha-Ara-Man-Glc-Gal (1.00:5.29:3.66:9.11:5.17)	—	Immunostimulant	Jiang et al. (2009), Zhou et al. (2009)
APF3	1.6 × 10^4^	Rha-Ara-Man-Glc-Gal (1.00:4.54:2.98:11.09:7.45)	—	Immunostimulant	Cao et al. (2008a), Jiang et al. (2009), Yang et al. (2008a)
APS-2a	7.4 × 10^5^	Glc-Gal-Ara-Rha-GalA (1.0:7.5:38.2:2.6:4.9)	I	Anti-tumor	Cao et al. (2010b)
ASP	I	Man-Rha-GlcA-GalA-Glc-Gal-Ara-Fuc (1.2:4.5:1.0:10.5:17.8:37.5:8.7:4.9)	—	Antioxidants	Sun et al. (2010)
ASP	5.0 × 10³	Rha-GalA-Glc-Gal-Ara (0.05:0.26:14.47:1.00:1.17)	—	Colonic localization Drug carriers	Zhou (2018)
APS-bII	1.3 × 10^4^	Glc-Gal-Xyl-Ara (8.4:2.7:1.8:1.0)	—	Antitumor	Zhang et al. (2016b)
APS-3a	5.9 × 10^5^	Glc-Gal-Ara-Rha-Man (3.2:1.7:2.5:1.3:1.0)	—	Antitumor	Liu et al. (2020b)
APS-3b	2.3 × 10^5^	Glc-Gal-Ara-Rha-Man (2.3:5.4:6.8:1.0:1.2)	—	Antitumor	Pu et al. (2016)
APS-3c	1.4 × 10^4^	Glc-Gal-Ara-Rha-Man-Xyl(6.3:4.7:6.7:6.5:1.6:1.0)	—	Antitumor	Pu et al. (2016)
APS-la	4.9 × 10^4^	Ara-Glc-Gal (27.7:15.0:57.3)	Mainly composed of 1,4-linked galactose, 1,3,6-linked galactose, T-galactose and T-arabinose	Antiradiation	Pu et al. (2016)
APS-3a	6.5 × 10^4^	Ara-Glc-Gal (6.5:9.0:84.5)	1,4-Gal, 1, 3,6-Gal, T-Gal and T-Ara	Antiradiation	Liu et al. (2022b)
SASP	8.1 × 10^4^	Glc-Gal-Ara (5.3:1.6:1)	Main chain (1→3)-linked Galp, (1→6)-linked Galp and 2-OMe-(1→6)-linked Galp composed of the C-3 position of the branched 2–0Me-(1→6)-linked Galp linked to the main chain	Antioxidant	Liu et al. (2022b)
JASP-1A	8.6 × 10^5^	Glc-Gal-Ara (1:4.55:4.92)	consisting of a 1,4-linked Galp and a 1,3-linked Rha, the branched-chain is linked to the C-6 position of the 1,4-linked Galp on the main chain, the terminal structure of the branched chain is T-Araf	Antioxidant	Liu et al. (2022b)
JASP-1B	7.2 × 10^4^	Man-Rha-GlcA-GalA-Glc-Gal-Ara (2.16:2.96:3.31:2.34:1:49.1:40.5)	—	Antioxidant	Liu et al. (2022b)
JASP-2	5.6 × 10^4^	Man-Rha-GlcA-GalA-Glc-Gal-Ara (3.32:7.88:4.79:15.9:0.5:46.6:42.1)	—	Antioxidant	Liu et al. (2022b)
Aps-2I	7.2 × 10^5^	Man-Rha-GalA-Glc-Gal-Ara (4:5:1:10:23:39)	—	Antioxidant	Liu et al. (2022b)
AAP-2A	2.2 × 10^5^	Rha-Gal-Ara-Glc (1:2.13:3.22:6.18)	1,3-Rhap, 1,3-Galp, 1,3-Araf, 1,5-Araf, 1, 3,5-Araf, 1,4-Glcp and 1, 4,6-Glcp	Antioxidant	Pantopoulos et al. (2012)

Note: - is not tested; Glc is glucose; Gal is galactose; Ara is arabinose; Rha is rhamnose; Fuc is fucose; Man is mannose; Xyl is xylose; GlcA is glucuronide; GalA is galacturonide; RhaA is rhamnose aldehyde.

### 2.4 Pharmacological effects of ASP

The medicinal use of *Angelica sinensis* was first reported by Shennong Ben Cao Jing, where it was considered to have the effect of nourishing the five viscera and regenerating muscles and later by researchers as a sacred medicine for blood (Pantopoulos et al., 2012). *Angelica sinensis* has a wide range of clinical applications since ancient times. Several researchers have conducted numerous studies on its components and pharmacological effects. ASP is one of the main active components of *Angelica sinensis*, and its extraction, purification, and mechanism of action have been studied in recent years. However, few in-depth studies have been conducted on its pharmacological effects. In this paper, the pharmacological effects of ASP were investigated, and it was concluded that the pharmacological effects of ASP primarily include improving anemia and antitumour effects and enhancing immune function, antioxidation, hepatoprotection, anti-inflammation, anti-fibrosis, hypoglycemia, antiradiation and antiviral properties.

#### 2.4.1 Improving anemia

ASP can alleviate anemia symptoms by increasing the levels of hemoglobin, red blood cells, erythropoietin, and iron-regulating hormones in the liver. Ferroregulin is a peptide hormone synthesized and secreted by the liver and is involved in iron regulation. Ferroregulin downregulates membrane iron transport proteins, inhibits the release of serum iron, and negatively regulates iron homeostasis in the body (Zhang and Wang, 2018).

Anemia caused by chronic disease (ACD) is secondary to chronic infection, inflammation, and tumors. In a study, ASP (0.5, 1 g/kg) was administered intraperitoneally to rats with ACD and was found to alleviate anemia by interrupting the IL-6/STAT3 (interleukin-6/signal transducer and activator of transcription 3), and BMP/SMAD (bone morphogenetic protein/SMAD) pathways to inhibit inflammatory iron-regulated proteins. In an animal experimental study, researchers constructed ASP-modified iron oxide nanoparticles (IONPs) and demonstrated the therapeutic effects of IONPs-ASP on iron deficiency anemia (IDA), which was associated with IONPs supplementation and APS-stimulated hematopoietic cell generation (Ma et al., 2024). Additionally, ASP promoted erythropoiesis through the IkB kinase-IkBα pathway (Wang et al., 2017b). Wang et al. (2018) showed that ASP (0.5, 1 g/kg) alleviates the inhibition of erythropoietin by increasing the protein expression of hypoxia-inducible factor-2α (HIF-2α) and decreasing the expression of inflammatory cytokines in rats with chronic renal anemia to achieve an anti-anemic effect.

In addition to chronic anemia, iron deficiency anemia (IDA) is also common. Relevant animal experiments suggested that after the treatment of ASP (0.3, 0.6, and 1.2 g/kg) for blood loss rats, it was found that the expression of Smad 1/5/8, Jak and ERK were inhibited, and then the expression of hepcidin was inhibited, so as to alleviate the symptoms of blood loss in rats (Zhang et al., 2014; Zhang et al., 2012b). A study examining the expression of hepatic-related proteins in rats with blood loss after ASP (1 g/kg) administration via gavage revealed that A inhibited the expression of hepatic (C/EBPα) (CCAAT/enhancer binding protein α), STAT3/5 and SM (Bi et al., 2021). The expression of AD4 stimulates the secretion of erythropoietin and alleviates IDA symptoms (Liu et al., 2012; Wang et al., 2011b). In a study conducted by Wang et al. (2017a), who injected ASP (4, 6 mg/kg) intraperitoneally into blood-lost mice, it was found that ASP increased the number of bone marrow stromal cells and increased the expression of intercellular adhesion molecule-1 (ICAM-1), thereby enhancing the proliferation ability of hematopoietic stem cells.

Additionally, bone marrow suppression caused by radiotherapy and chemotherapeutic agents can cause anemia. ASP (10 mg/kg) was administered to mice after X-ray radiation and was found to inhibit cellular senescence through the phosphatidylinositol-3-hydroxyl kinase/protein kinase B (PI3K/Akt) pathway, promote the repair of platelets, blood cells, and progenitor cells, and alleviate anemia (Liu et al., 2010). He et al. (2012) reported that ASP (2, 8 mg/kg) decreased the percentage of apoptotic peripheral red blood cells, white blood cells, and bone marrow cavity blood cells in irradiated mice. These findings suggest that ASP can protect against radiation injury by altering microRNA expression in blood and restoring the bone marrow hematopoietic system in radiation-irradiated mice (Li and Xu, 2017). ASP (50, 200 mg/kg) also promoted the recovery of peripheral blood leukocyte counts and the transformation of splenic lymphocytes (Sun et al., 2009), enhancing radiation tolerance in mice. Radiation-exposed mice injected with ASP (0.2 mL/piece, once a day) had significantly reduced hematocrit and cellular damage caused by radiation (Hong et al., 2002). Through preclinical and cellular studies, researchers have shown that the polysaccharide components of traditional Chinese medicine have protective effects on the hematopoietic system. Ding Xuelan’s team administered ASP (100 μg/g, 200 μg/g, and 400 μg/g) by gavage to mice with cyclophosphamide-induced myelosuppression. The levels of red blood cells, white blood cell platelets, immunoglobulin G (IgG), and immunoglobulin M (IgM) were significantly increased, and the enhanced immunomodulation may act with cytokines on various lineages of hematopoietic progenitor cells, hematopoietic stem cells, and lymphocytes to promote the recovery of the hematopoietic system (Ding et al., 2016).

Lee et al. (2012) fractionated ASP (6,000 g/mL) with a DEAE-Sepharose CL-6B column and obtained four fractions (F1, F2, F3, and F4), of which the F2 fraction was found to have the highest hematopoietic activity by magnetically activated cell sorting. F2 accounted for 19% of the ASP and 0.53% of the protein content. The monosaccharide components of F2 were arabinose (51.82%), fructose (1.65%), galactose (29.96%), glucose (4.78%), and galacturonic acid (14.80%). It stimulates the secretion of granulocyte macrophage-colony stimulating factor (GM-CSF) and IL-3 by human peripheral monocytes and protects the hematopoietic function of CD34^+^ cells with strong hematopoietic activity.

#### 2.4.2 Antitumor

Tumors are formed by the proliferation of local cells in response to various carcinogenic factors. Some studies have shown that iron overload increases the incidence of cancer (Basak and Kanwar, 2022). Tumors can be treated by several methods, such as increasing the body’s immunity, inducing tumor cell differentiation, promoting tumor cell apoptosis, and reducing iron content (Dienstmann and Tabernero, 2017). The antitumor activities of ASP and the underlying mechanisms are summarized in Table 2.

**TABLE 2:** Antitumor effects and mechanisms of ASP.

Types of cancer	Pharmacological action	Pathways/molecules involved	Models	References
Leukemia	Inhibits tumor cell proliferation; induces tumor cell differentiation and promotes cellular senescence; increases the toxicity of natural killer cells to tumor cells	JAK2/STAT5 pathway/TNF-α, IL-2, IFN-γ	*In vivo*: DBA/2 mice *in vitro*: K562 cells, L1210 cells	Liu et al. (2019), Wang et al. (2015b), Zhang et al. (2016b)
Liver cancer	Inhibits ferroregulation, thereby inhibiting the growth and proliferation of tumor cells; directly inhibits tumor cell proliferation	JAK/STAT and BMP-SMAD pathways/IL-6, JAK2, STAT3, SMAD1/5/8, ferritin, Tf, TfR1, TfR2	*In vivo*: BALB/c mice; *in vitro*: HHCC cell line A549 cells, HepG2 cells, H22 cells	Ren et al. (2018), Shang et al. (2003)
Breast cancer	Inhibits tumor cell metastasis; induces apoptosis in tumor cells and reduces ferroregulation	CREB and JAK/STAT, BMP-SMAD pathway/Caspase, PARP, Bax, Bcl-xl, Apafl, IL-6, STAT3, SMAD1/5/8, etc.	*In vivo*: Balb/c mice; *in vitro*: T47D cells, MCF-7 cells, 4T1 cells	Ren et al. (2018), Zhou et al. (2015)
Cervical cancer	Inhibits tumor cell proliferation and promotes apoptosis of tumor cells	Caspase-3/9, PARP, p-p38, MMP-2 and MMP-9	*In vivo*: Balb/c mice *in vitro*: HeLa cells	Cao et al. (2010a), Tang et al. (2020)
Neuroblastoma	Inhibits tumor cell proliferation and migration; induces apoptosis	PI3K/AKT and JAK/STAT pathways/Bcl-2, Bax, Caspase-3/9	*In vitro*: SH-SY5Y cells	Yang et al. (2018)

Leukemia is a malignant hematological tumor caused by the diffuse malignant growth of a certain type of immature leukocyte in the bone marrow that replaces normal bone marrow tissue and diffuses into the bloodstream and lymphatic system. Currently, the conventional treatments for leukemia include chemotherapy, radiotherapy, targeted therapy, bone marrow transplantation, and supportive therapy. Liu et al. (2019) reported that leukemic mice treated with different doses of ASP (12.5 μg/mL, 25 μg/mL, 50 μg/mL, 100 μg/mL) exhibited prolonged survival to varying degrees, and the leucocyte, lymphocyte, tumor necrosis factor-α (TNF-α), interleukin 2 (IL-2), and interferon-gamma (IFN-γ) levels increased significantly. The findings of this study suggest that ASP may suppress leukemia by enhancing specific and nonspecific immunomodulatory functions. In addition, one study revealed that ASP (12.5 μg/mL, 100 μg/mL) promotes ASP-induced differentiation of leukemia cells by inducing JAK2/STAT5 tyrosine phosphorylation and activating erythropoietin (EPO) (Wang et al., 2015b). *In vitro* experiments have shown that (Zhang et al., 2016b) ASP (100, 200, 300, 400, and 500 mg/L) can inhibit the proliferation of leukemic cells, promote the proliferation of splenocytes, and enhance macrophage and phagocytic activity and the cytotoxicity of natural killer cells.

Liver cancer is a malignant tumor with a high mortality rate. An *in vitro* study of H22 cells incubated with ASP showed that low doses of ASP promoted the proliferation of splenic lymphocytes, inhibited tumor growth, and significantly inhibited tumor growth by reducing iron concentrations in the liver, spleen, and tumor cells (Cheng et al., 2016). ASP (30, 100 and 300 mg/kg) can inhibit the invasion and metastasis of hepatocellular carcinoma cells *in vitro* (Shang et al., 2003). Several studies have shown that the expression levels of IL-6, STAT3, JAK2, SMAD1/5/8, ferritin, the transcription factor TfR1 (transferrin receptor 1), and TfR2 (transferrin receptor 2) are reduced in the liver tissues of mice with hepatocellular carcinoma after the administration of ASP (100 mg/kg) (Ren et al., 2018).

Breast cancer occurs frequently in the female population, is a common malignancy, and has multiple complex mechanisms. A study in which breast cancer was induced in nude mice by injecting breast cancer cells followed by intraperitoneal administration of ASP (0.2 mg/kg) showed that the cAMP-responsive element-binding protein cyclic adenosine effector element binding protein (CREB) (Zhou et al., 2015), poly ADP ribose polymerase (PARP), Caspase-3, Caspase-9, myeloid leukemia gene 1 (Mcl-1), Bcl-2, Bcl-xL and apoptosis-activating factor 1 (Apaf1) were upregulated, resulting in increased tumor cell apoptosis. Another study showed that ASP (100 mg/kg) inhibits tumor growth by decreasing the expression of ferro regulatory elements (Ren et al., 2018). Furthermore, by culturing breast cancer cells *in vitro*, ASP (100, 200, 300, 400, and 500 mg/L) was found to exhibit antitumor activity by directly inhibiting cancer cell proliferation (Zhang et al., 2016b).

Cervical cancer is a common gynecological malignancy, and its incidence has recently been reported in younger populations. In a study performed on cervical cancer-induced nude mice, after ASP treatment, the mitochondrial potential was reduced, and caspase-9, caspase-3, and PARP expression was greatly increased, indicating that ASP (3, 30 or 300 mg/mL) inhibits tumor proliferation in cervical cancer by activating mitochondrial apoptosis (Cao et al., 2010a). In addition, a dose-dependent ASP (100, 200, and 400 mg/L) study showed inhibition of the growth and migration ability of HeLa cervical cancer cells (Tang et al., 2020), a reduction in the scratch closure rate and number of invasive cells, and downregulation of p-p38, MMP-2, and MMP-9 protein expression. This inhibitory effect may be achieved in conjunction with the influence of MMP-2 and MMP-9 expression by modulating p38 signaling pathway activity.

Neuroblastoma occurs frequently in children and infants and has a high recurrence rate and poor prognosis. *In vitro* culture of neuroblastoma cells treated with ASP (100, 200, 300, 400 or 500 ug/mL) revealed that ASP downregulated NA-H19 expression in neuroblastoma cells and that miR-675 upregulated CD44 expression and may prevent the formation of neuroblastoma tumor cells by inhibiting the miR-675-mediated PI3K/AKT and JAK/STAT pathways (Yang et al., 2018).

#### 2.4.3 Antioxidants

Free radicals are highly biologically active and oxidizing compounds produced by metabolic reactions in the body using oxygen. Under normal circumstances, the body maintains a dynamic balance between oxidation and antioxidation; however, when free radicals or the antioxidant capacity of the body is unbalanced, the body can damage cell structure, protein, and nucleic acid, thus accelerating aging and even inducing various diseases. Recent research has shown that ASP has a strong scavenging effect on free radicals in the body, regulating phenol oxidase (PO), superoxide dismutase (SOD), and glutathione peroxidase (GSH-PX) activities, reducing malondialdehyde (MDA) and alleviating the oxidative stress response of the body (Cao et al., 2018). ASP can protect the body from oxidative stress by improving the antioxidant capacity of body tissues; it is also important for enhancing beauty and delaying aging. Studies have shown that ASP can reduce damage to cellular oxidative reactions caused by H_2_O_2_, improve cell viability, and attenuate apoptosis and ROS production. In the H_2_O_2_-induced cardiomyocyte H_9_c_2_ oxidative stress model, APS (6.25, 12.5, 25, 50, and 100 mg/mL) exerts protection against H9c2 by reducing endoplasmic reticulum (ER) stress and oxidative stress, alleviating H_2_O_2_-induced cytotoxicity and apoptosis through activation of the ATF6 pathway (Niu et al., 2018). The antioxidant effects of ASP are shown in Table 3.

**TABLE 3:** Antioxidant effect of ASP.

Physiological or pathological state	Subjects	Efficacy	Drug dosage	References
Arthritis	Human chondrocytes	Reduces oxidative stress and protects chondrocytes	10 mg/kg	Liu et al. (2020c)
	Macrophages isolated from the peritoneal cavity of BALB/c mice	Protective and antioxidant effects on H_2_ O_2_ damaged macrophages	5, 25, 125 μg/mL	Zhuang et al. (2016)
Healthy organism	Middle-aged healthy women	Reduces oxidative stress and stimulates the expression of antioxidant enzymes	125 mg/kg	Jiang et al. (2009)
Arthritis	Rat chondrocytes	Reduces damage to mouse chondrocytes by H_2_O_2_, promotes cellular secretion of antioxidant enzymes, and repairs cellular damage	10 mg/kg	Liu et al. (2020c)
Cervical cancer	HeLa cells	Protective effect against oxidative damage to cells caused by H_2_ O_2_ or UV irradiation	0.3, 1, 3, 10, 30, 100 μg/mL	Jia et al. (2007)
Healthy organism	Shrimp	Increased levels of phenol oxidase activity, superoxide dismutase activity, glutathione peroxidase	0.5, 1,1.5 g/kg	Pan et al. (2018)
Iron deficiency anemia	Rats	Increased serum superoxide dismutase and glutathione peroxidase activities and decreased MDA in rats with iron deficiency anemia	15 mg/kg, 30 mg/kg, 60 mg/kg	Wu et al. (2017)
Carbon tetrachloride causes liver damage	Mice	Mechanism of attenuating or even reversing CCl4-induced hepatic oxidative damage in mice is associated with increased lipid peroxidation and enhanced antioxidant enzyme activity	150 mg/kg, 300 mg/kg	Yu et al. (2013)

#### 2.4.4 Immunomodulation

The immune system, which is composed of various components, protects the body by acting as an effective barrier against pathogenic invasion. ASP improves nonspecific immunity by enhancing immune cell activity. South American white shrimp were fed ASP (0.5, 1,1.5 g/kg) for 12 weeks and showed enhanced nonspecific immunity, increased survival, and increased resistance to *Vibrio* lysis (Pan et al., 2018). *In vitro* culture of mouse peritoneal macrophages revealed that ASP (20, 25, 50, 100, and 200 μg/mL) promoted macrophage proliferation, enhanced macrophage activity by modulating lysozyme activity, and increased the expression of H_2_O_2_, NO, TLR4 (toll-like receptor family 4), and ICAM-1 (Chen et al., 2010). ASP (10, 30, and 100 μg/mL) can enhance nonspecific immunity in addition to specific immunity. In a study conducted on murine leukemia virus (Yang et al., 2006), the percentage of CD4^+^ cells and the CD4^+^/CD8^+^ ratio in peripheral blood cells were found to be significantly increased by ASP at 3–30 mg/kg. It has also been demonstrated that ASP (3, 10, and 30 mg/kg) has immunomodulatory activity and is able to promote the proliferation of T cells (Yang et al., 2012), increase the production of IL-2 and IFN-γ, and decrease the production of IL-4 to regulate the expression of Th1- and Th2-related cytokines. Lu et al. (2017a) administered ASP (254.2 mg/kg) by gavage to rats exposed to whole-body X-ray radiation and observed a significant increase in the serum levels of IL-4 and IFN-γ, indicating that ASP has immunoprotective effects on X-ray radiation. A study conducted on ulcerative colitis-induced mice revealed that treatment with ASP (0.5 g/kg, 1 g/kg) reduced inflammation by decreasing the cytokine IL-10 (Pan et al., 2015). Ding et al. (2016) studied the effects of different doses of ASP (100, 200, and 400 ug/g) on myelosuppression in mice and reported that ASP increased the levels of IgG, IgM, and T-cell subsets, enhancing both humoral and cellular immunity.

#### 2.4.5 Hepatoprotective effect

**FIGURE 4:**
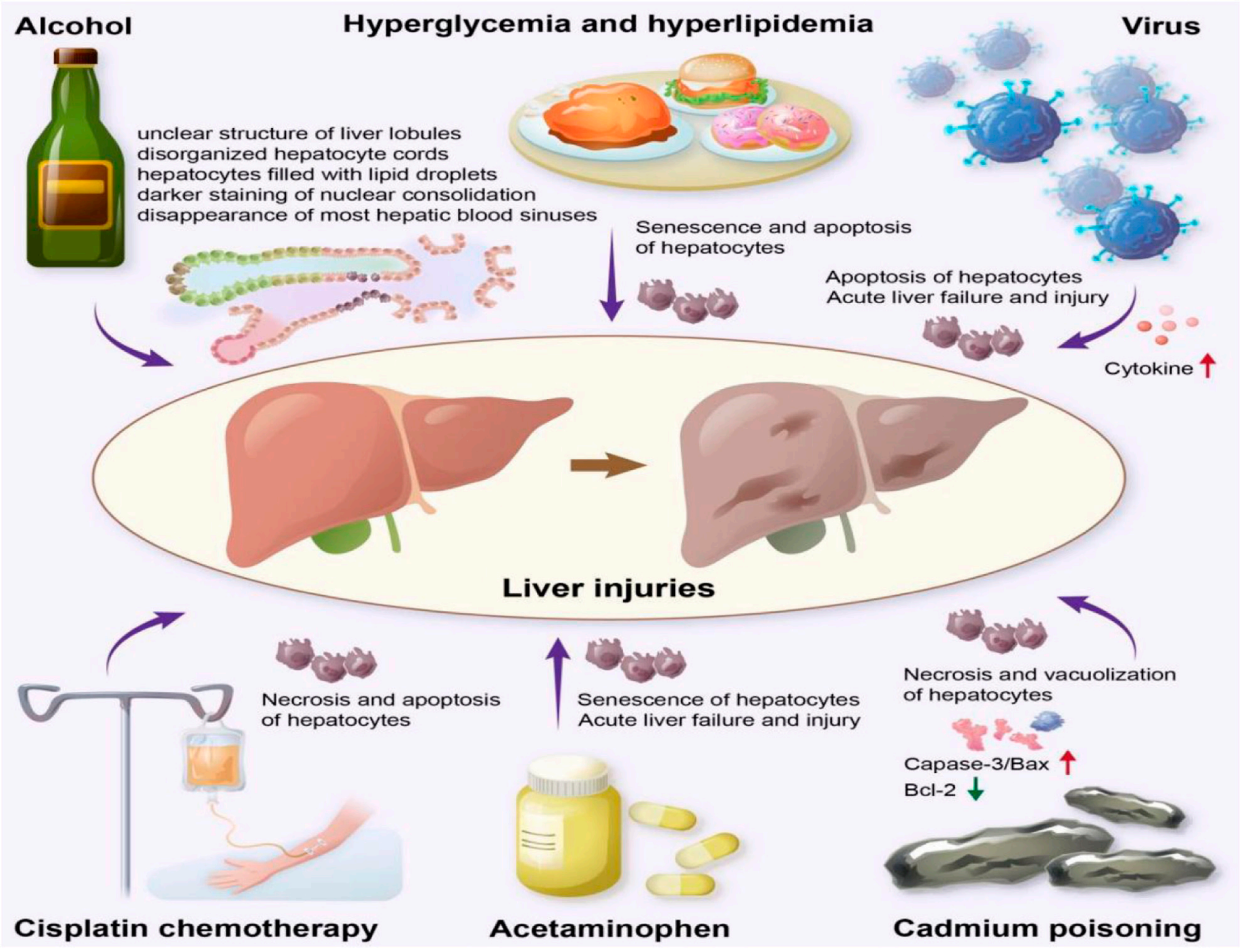
Various factors that can cause liver damage.

The liver is a crucial organ of the body and is involved in physiological functions, such as synthesis, metabolism, excretion, detoxification, and immunity. The incidence of liver injury, which seriously threatens human health, is increasing annually. It has been reported (Horvatits et al., 2019) that factors such as alcohol consumption, drug abuse, and viral infection can damage hepatocytes, leading to body imbalance and impaired energy regulation, which causes liver injury (Figure 4). ASP has shown protective effects against various types of liver injuries, and the mechanisms involved are reviewed below.

Alcoholic liver injury is a disease caused by long-term alcohol consumption, the incidence of which is increasing annually, and it is the second most common liver disease after viral hepatitis. Alcohol-induced liver injury is characterized by an unclear structure of the liver lobules, disorganized hepatocyte cords, hepatocytes filled with lipid droplets in the form of vacuoles, darker staining of nuclear consolidation, and the disappearance of most hepatic blood sinuses (Wang et al., 2016a). ASP (4 mg/kg) increases the activity of antioxidant enzymes, scavenges oxygen free radicals, and reduces the chain reaction of membrane lipid peroxidation, which subsequently restores the biofilm structure of hepatocytes in alcohol-induced liver injury (Jia et al., 2015). ASP (1.5 mg/kg, 6 mg/kg) alleviates viral and autoimmune hepatitis, reduces cytokine expression and inflammatory responses in experimental mice, and it may inhibit hepatocyte apoptosis by attenuating the c-Jun amino-terminal kinase (JNK)-mediated mitochondrial apoptotic pathway and inhibiting Caspase-8 expression (Wang et al., 2016c).

In addition to alcoholic liver injury and viral hepatitis, liver injury caused by drugs and cadmium poisoning is also common, with acetaminophen (APAP) being the most common cause of liver injury (Han et al., 2010). Long-term or excessive use of APAP is associated with acute liver failure and injury. Cao et al. (2018) investigated the reduced toxicity of different concentrations of ASP (3, 6, and 12 mg/kg) intravenously administered for 10 days in APAP-induced liver injury rats, and APAP-induced histological changes were significantly reversed with vacuolization and necrosis of the cytoplasm. In addition, the expression of caspase-3 and Bax decreased, the expression of Bcl-2 increased, and hepatocyte apoptosis was inhibited. Liu et al. (2018) showed that ASP (20 mg/kg) had a significant protective effect on liver injury in cadmium-treated rats. After treatment with ASP, the thymus, spleen index, lymphocyte transformation capacity, NK cell killing capacity, cytokine IL-2 content, and Bcl-2 protein expression were significantly increased in cadmium-treated rats, while AST, ALT, LDH activity, TGF-β1, and cadmium levels were increased. This mechanism may involve the enhancement of autoimmunity, the regulation of enzyme activity, and the expression of apoptosis-related proteins.

Furthermore, chemotherapeutic drugs used to treat tumors are associated with apoptosis and necrosis of liver cells. Fu and Ma (2018) reported that ASP (25, 50, and 100 mg/kg) could improve liver function by increasing the number of leukocytes, erythrocytes, and platelets, which decreases the level of Bax and increases the expression of Bcl-2. This results in alleviating liver injury induced by cisplatin in H22 ascites-derived tumors in mice.

Hyperglycemia and hyperlipidemia can cause liver damage. Kaiping Wang et al. (2016d) induced hyperglycemia and hyperlipidemia in mice using a high-fat diet and low-dose streptozotocin (STZ) and observed that the rats developed significant liver damage. During the experiment, the mice were administered different doses of ASP (100, 200 and 400 mg/kg) along with STZ injection. ASP has been found to reduce liver damage by stimulating insulin secretion, facilitating hepatic glycogen synthesis, releasing adipokines, and reducing hepatic fat accumulation. In addition, ASP promoted the expression of the antiapoptotic protein Bcl-2 and reduced the expression of the proapoptotic protein Bax, which inhibited the senescence of hepatocytes and reduced liver injury in mice.

#### 2.4.6 Anti-inflammatory effects

Experiments have confirmed that ASP has therapeutic effects on several inflammatory conditions, such as osteoarthritis and colitis. Chondrocyte apoptosis is important in the development of osteoarthritis (OA). Xu et al. (2021) reported that ASP (50 ug/mL, 200 ug/mL) had a protective effect against sodium nitroprusside (SNP)-induced chondrocyte apoptosis, suggesting that ASP is a potential alternative for OA treatment. Xu et al. (2022) performed a study on an *in vitro* model of IL-1β injury and observed that ASP (10, 50, and 100 ug/mL) effectively reduced IL-1β damage to chondrocytes and increased their activity. Additionally, ASP reduced the expression levels of β-catenin, Wnt4a, GSK-3β, MMP-13, and ADAMTS4, inhibited IL-1β-induced cartilage degradation, and alleviated patient symptoms. ASP has been found to inhibit oxidative stress damage and inflammatory responses in osteoarthritic chondrocytes through the Wnt/β-catenin signaling pathway. Preclinical studies have shown that it (0.5 g/kg, 1 g/kg) significantly reduces the production of IL-6, TNF-α, and other proinflammatory factors and has a significant therapeutic effect on arthritic rats (Li et al., 2020).

Cheng et al. (2020) reported that ASP (200 mg/kg) reduced myeloperoxidase activity in colonic tissues, modulated the expression of proinflammatory cytokines and related proteins, and improved dextran sodium sulfate-induced colitis in mice. It has also been reported that ASP (200 mg/kg) significantly reduces the serum expression levels of IL-6, TNF-α, SDF-1, plasma TF, D-D, and FIB in rats prone to embolism during pregnancy (Ma et al., 2022), indicating that APS has anti-inflammatory and anticoagulant effects. *In vitro* studies have shown that ASP (80 ug/mL, 100 ug/mL) can reduce the inflammatory response to protect neuronal cells (Zhou et al., 2019). Zhou et al. (2018) analyzed the effects of ASP (0.009, 0.018, and 0.036 g/mL) on TLR4/MyD88/NF-κB pathway inhibition in rats with diabetic peripheral neuropathy (DPN) and reported that APS significantly decreased the levels of IL-6, myelin basic protein (MBP), TNF-α, CRP, NF-κB, MyD88, and TLR4 and significantly increased CAT mRNA expression in rats, reduced the levels of inflammatory factors, and decreased damage to the inflammatory response.

#### 2.4.7 Anti-fibrosis

Wang et al. (2010) observed the effects of ASP (50, 100, and 200 g/kg) on pulmonary function and the lung coefficient in rats with pulmonary fibrosis induced by pulmonary fibrosis and reported that 0.3 s of expiratory volume as a percentage of forced expiratory volume (FEV0.3/FVC) and calm end-expiratory function residual capacity (FRC) increased to different extents, and it was shown that ASP could significantly improve pulmonary function, increase body weight, and decrease the lung coefficient in rat models of pulmonary fibrosis. Qian et al. (2020) reported that ASP (20 mg/kg) inhibited the development of fibrosis in rat lung and alveolar type II epithelial cells (RLE-6TN) through the DANCR/AUF1/FOXO3 regulatory axis, which facilitates the treatment of idiopathic pulmonary fibrosis (IPF). In addition, ASP (12.5, 25, and 50ug/mL) was found to inhibit pulmonary fibrosis, and its effects may be related to the downregulation of α-smooth muscle actin (α-SMA) and CTGF expression and the regulation of the balance between MMP-9 and TIMP-1 (Luo et al., 2017). Song et al. (2021) reported that ASP (80, 160, and 320 mg/kg) decreased the LV end-diastolic diameter, LV end-systolic diameter, LV end-diastolic volume, and LV end-systolic volume; decreased the expression levels of TGF-β1, α-SMA, type I collagen (CoI), fibronectin, vimentin, Bax, cleaved caspase-9, and cleaved caspase-3 in the LV; increased SOD and GSH-Px activities; decreased MDA, H2 O2, and ROS levels; and ultimately inhibited myocardial apoptosis and oxidative stress to prevent hypertensive myocardial fibrosis in HHD rats by increasing the ejection fraction and fractional shortening. Another study showed that ASP (200 mg/kg) improved chronic liver fibrosis by inhibiting HSC activation through the IL-22/STAT3 pathway (Wang et al., 2020), reducing serum alanine aminotransferase by approximately 50%, and inhibiting hepatic stellate cell (HSC) activation in mice with liver fibrosis.

#### 2.4.8 Lowering blood sugar

Non-starch polysaccharides lower blood glucose levels through multiple pathways. It was found that after 4 weeks of ASP4 (100, 200, 400, and 600 mg/kg) treatment in STZ-induced diabetic mice (Wang et al., 2015a), fasting blood glucose (FBG) decreased, dyslipidemia improved, and elevated serum total cholesterol (TC) and triglyceride (TG) levels decreased. Zhang et al. (2019) reported a protective effect of ASP on the islets of T2DM mice, and the possible mechanism was related to the promotion of insulin secretion and the inhibition of apoptosis by blocking both internal and external pathways of pancreatic β-cells. In another study, Chen (2010) reported that after oral administration of ASP (2 or 100 mg/kg) for 21 days, alloxan-induced diabetic rats exhibited significantly decreased blood glucose levels and improved plasma insulin levels, which may be associated with the repair and regeneration of damaged pancreatic β-cells. Several studies have shown that ASP (80, 160 and 320 mg/kg) reduces blood glucose levels and attenuates insulin resistance by modulating relevant metabolic enzymes and activating the PI3K/Akt pathway in high-fat diet-fed mice (Wang et al., 2016b), suggesting that ASP has therapeutic potential for treating hypoglycemia.

ASP-1I (12.5, 25, and 50 mg/kg) has been shown to enhance glucose absorption while simultaneously inhibiting the activation of p-IRS-1, p-IRS-2, p-JNK, and p-P38 pathways in insulin-resistant HepG2 cells. Furthermore, intraperitoneal administration of ASP-1I significantly ameliorates insulin resistance and suppresses the RAGE-JNK/P38-IRS pathway in the liver of diabetic rats, demonstrating a mild yet effective therapeutic effect (Liu et al., 2022a). Additionally, oral administration of ASP (40 mg/L) can improve bone health by reducing blood glucose levels and enhancing insulin sensitivity, thereby facilitating bone tissue repair in a rat model of type 2 diabetes (Liao et al., 2019).

#### 2.4.9 Radiation resistance

Currently, ionizing radiation has become the fourth major hazard to humans after air, water, and noise pollution. Polysaccharides are more suitable alternatives to medicinal radiation protection agents because they are less toxic and non-accumulative. ASP (63.5, 127, and 254 mg/kg) is used to protect against radiation-induced liver damage because of its ability to increase the resistance of the liver to radiation and to scavenge free radicals (Wang et al., 2017c). ASP (63.6, 127.1, and 254.2 mg/kg) regulates the body’s oxygen radical balance through activation of the transcription factor Nrf2, thereby counteracting radiation-induced oxidative damage (Lu et al., 2017b; Zhang et al., 2017). ASP (63.5, 127, and 254 mg/kg) has also shown efficacy against radiation-induced bone marrow and spleen damage in SD rats, probably by reducing radiation-induced damage to hematopoietic and immune cells in the bone marrow and spleen (Lu et al., 2017a; Xu et al., 2017). Zhang et al. (2020) showed that medium (127.1 mg/kg) and high (254.2 mg/kg) doses of ASP had significant effects on ionizing radiation-induced intestinal barrier damage in SD rats. ASP (100, 200, and 400 mg/kg) can regulate the ratio of regulatory cells (Tregs)/helper T cells 17 (Th17) in ^60^Co-γ-irradiated mice, improve the stability of mitochondrial membranes, and regulate abnormal levels of ROS and mitochondria-related apoptotic proteins, thus achieving antiradiation effects (Chen et al., 2022).

### 2.5 Other pharmacological effects

Studies have shown that ASP can lower blood lipids, improve diabetic nephropathy, and improve antiviral properties.

ASP (400, 600 mg/kg) significantly reduced the homeostasis model assessment-insulin resistance index (HOMA-IR) and body weight and was also effective in reducing serum TC and triglyceride concentrations and restoring pancreatic/liver or adipose tissue in BALB/c mouse models of prediabetes and STZ-induced diabetes (Wang et al., 2015a).

A major complication in long-term diabetic patients is diabetic nephropathy (DN). Intraperitoneal injection of the ASP branch Acanthopanax (AG) for 8 weeks in diabetic rats resulted in significant improvements in renal function, increased creatinine clearance, a significant reduction in blood urea nitrogen, and the expansion of glomeruli in patients with proteinuria. AG (20, 50, and 100 mg/kg) inhibited the RAGE/NF-KB (receptor for advanced glycosylation end products/nuclear factor-KB) signaling pathway by inhibiting the over-proliferation of glomerular mesangial cells (GMCs) via the RAGE/NF-KB signaling pathway and attenuating inflammatory mediators (Sui et al., 2019).

ASP (1.5 g/kg) was found to promote the maturation of dendritic cells (DC) in hepatitis B virus (HBV) transgenic mice, upregulate the expression of the surface costimulatory molecule CD86, improve the ability of DC to proliferate lymphocytes and secrete IL-12 and r-IFN, and may play a critical role in the antiviral immunity of HBV transgenic mice (Li et al., 2005). APS has shown a synergistic effect with dithiothreitol, suggesting that APS can be used in combination with antiviral drugs for the treatment of AIDS. ASP (10, 30 mg/kg) causes anti-cellular oxidative damage, which may be the mechanism associated with anti-AIDS activity (Jia, 2005). ASP have shown efficacy against human cytomegalovirus (HCMV)-infected cytomegalic lineage cells *in vitro* by inhibiting HCMV-induced apoptosis in a dose-dependent manner (Zhang et al., 2009). ASP enhance the proliferation of chicken embryonic fibroblasts and prevents infection by Newcastle disease virus (Hu et al., 2004). The chlorosulfate-pyridine-modified *Angelica* sulfated polysaccharides (0.244, 0.488, 0.977, 1.953, and 3.907 ug/mL) significantly improved resistance to Newcastle disease virus infection in chicken embryo fibroblasts (Wang et al., 2011a). An *in vitro* study demonstrated that ASP (1,399.531 ug/mL) inhibited chicken infectious bursal virus-infected cells in a quantitative and temporal manner (Hu et al., 2003).

*Angelica sinensis* polysaccharide, as a traditional Chinese medicine component, has shown significant antiviral potential in pharmacology, which has been widely recognized in the scientific community. However, any medicinal ingredient has its two sides, angelica polysaccharide is no exception. Studies have shown that angelica polysaccharide can effectively promote the proliferation of fibroblasts, which has a positive effect on the repair of damaged tissues and wound healing. Fibroblasts are the main cell type that constitutes connective tissue, and their proliferation is essential for maintaining the integrity and elasticity of skin and tissue. However, in some cases, excessive proliferation of fibroblasts can lead to undesirable side effects, such as excessive scar tissue formation during healing, and may even cause tissue sclerosis, affecting organ function. Therefore, when considering the use of ASP for treatment, we must carefully weigh its potential benefits against possible risks to ensure that unnecessary side effects are avoided while promoting health.

### 2.6 Effect of different extraction positions of *Angelica sinensis* on ASP activity

*Angelica sinensis*, a traditional Chinese herbal medicine, shows unique differences in pharma cological effects of polysaccharide components contained in different parts. The content of ASP in *Angelica sinensis* is as high as 15% (Cao, 2019). As one of the main components of *Angelica sinensis* for nourishing blood and regulating menstruation (Ouyang et al., 2005), it can improve the hematopoietic function of the body by increasing the number of white blood cells, red blood cells and hemoglobin in peripheral blood. The study of Wu Guoxia et al.believed that in different medicinal parts of *Angelica sinensis*, the amount of ASP contained in *Angelica sinensis* was significantly higher than that in *Angelica sinensis* head and other parts (Wu et al., 2018a). Song et al. also confirmed the view that the content of ASP in *Angelica sinensis* was higher by investigating different cultivation methods of *Angelica sinensis* (Song et al., 2008). By comparing the content of ASP in the whole *Angelica sinensis* and the head of *Angelica sinensis* (Long and Maobao, 2008), think that the content of ASP in the whole angelica processed in the same origin is higher than that in the head of *Angelica sinensis*; considering that the whole *Angelica sinensis* and *Angelica sinensis* can be clustered into one class in the process of fingerprint cluster analysis (Wu et al., 2018b), we can still think that the polysaccharide content in *Angelica sinensis* is higher than that in *Angelica sinensis* head and *Angelica sinensis* tail, and is close to that in *Angelica sinensis*.

Some scholars have studied the antioxidant activity of polysaccharides from different medicinal parts of *Angelica sinensis* (Zou et al., 2022). Four kinds of polysaccharides, ASP-H-AP, ASP-B-AP, ASP-T-AP and ASP-Hb-AP, were obtained from different medicinal parts of *Angelica sinensis*, and their antioxidant activities were studied. It was found that the four polysaccharide components could reduce the oxidative stress of IPEC-J2 cells by up-regulating the expression of related genes and proteins of antioxidant enzymes. Among them, ASP-Hb-AP had better antioxidant effect, while ASP-T-AP had relatively poor antioxidant effect.

ASP in *Angelica sinensis* can induce the synthesis and secretion of hematopoietic regulatory factors by macrophages by enhancing the expression of GM-CSF protein and mRNA in bone marrow stromal cells, spleen cells and thymocytes, and accelerate the proliferation and differentiation of myeloid multi-directional hematopoietic progenitor cells, late erythroid progenitor cells and granulocyte mononuclear hematopoietic progenitor cells. Finally, it can restore the number of peripheral blood cells in blood deficiency animals, reconstruct the long-term hematopoietic ability of hematopoietic failure animals (Xu et al., 2020), improve the immune adhesion ability and hematopoietic ability of red blood cells after radiation injury, and improve anemia. In addition, it also has the effects of promoting blood circulation, hemostasis, anti-radiation, anti-oxidation, anti-tumor, anti-inflammation and improving immunity.

On the other hand, the pharmacological effects of polysaccharides in the head and tail of *Angelica sinensis* have not been reported in domestic and foreign studies, and further research and exploration by the research group are needed.

### 2.7 Summary and outlook

In summary, ASP is the main active component of the traditional Chinese medicine *Angelica sinensis*, which has multiple components, multiple target characteristics, and very broad pharmacological activities. It also plays an important role in the treatment of several diseases. In this review, we describe the ten major pharmacological activities of ASP reported in several studies, including anemia, antitumour, antioxidant, immunomodulatory, hepatoprotective, anti-inflammatory, hypoglycemic, antiradiation, antifibrosis, and antiviral activities. However, preclinical studies on ASP have primarily focused on crude compounds without further purification and isolation, which necessitates further investigation to determine the specific composition of ASP. Studies have shown that ASP modification helps amplify their pharmacological effects (Hou et al., 2021). Therefore, studying the effects of different modifying agents on the pharmacological activities of ASP is promising. ASP has been reported to play a crucial role in the treatment of diabetes mellitus and its complications, such as diabetic nephropathy and peripheral neuropathy. The incidence of diabetes mellitus is increasing annually worldwide, and patients require long-term medication. In such cases, the use of ASP as a natural plant extract with fewer side effects and a high safety profile is a potential alternative for the long-term treatment of diabetes mellitus. ASP has shown efficacy against tumors and organ fibrosis; however, its specific molecular mechanisms of action and drug targets remain undetermined. Therefore, in-depth studies are required to identify the underlying mechanisms and potential drug targets involved.

As research on ASP has advanced, the use of ASPs in clinical, food, healthcare, and cosmetic applications has also gradually increased. Iron ASP have been used for the treatment of iron deficiency anemia (Wang et al., 2007b), sulfated ASP have been used as antitumour agents (Wu et al., 2012; 2013), and *Angelica* hepatica capsules have been used to lower blood sugar (Wang et al., 2007a; Zhang et al., 1998). In addition, *Angelica sinensis* and *Astragalus* iron particles and *Angelica Sinensis* and *Astragalus* capsule have been developed for iron deficiency anemia and prevention of radiation exposure, as shown in Table 4. However, more research and development are required to identify the various natural compounds and their contents or ratios. Standardized norms have not yet been developed, and the material basis of the effect and its target of action remain unclear. Studies on the use of dosages are not well standardized or precise. Therefore, a systematic evaluation of its safety and efficacy is needed. Standardization of the extraction process for specific active ingredients and identification of their precise targets are key issues that need to be addressed.

**TABLE 4:** *Angelica sinensis* polysaccharidederived health food.

Product name	Manufacturer	Treatment indications	Dose/formula	Regulatory status (CFDA)
*Angelica sinensis*, *Astragalus* and iron particles	Shenzhen McKinley Co., Ltd	Hypoferric anemia; iron-deficiency anemia	5 g/bag, containing 352 mg of iron, 60 mg of ASP, AS-IV 30 mg	National food health word: G20230585
*Angelica Sinensis* and *Astragalus* capsules	Gansu LongShen RongFa Pharmaceutical Co., Ltd	It has the auxiliary protection function for the radiation hazard	0.36 g/grain, containing 0.5 g of crude polysaccharide per 100 g	National food health word: G20140331

However, natural products often face challenges related to repeatability, standardization, and quality control. The repeatability, standardization, and quality control of ASP is also a challenge, but can be overcome through a series of scientific and rigorous methods and strict standards. Firstly, the active ingredient standard (ASP) was used as a reference to ensure that the content and quality of ASP in the product were consistent. This process involves precise chemical analysis and quality control techniques to ensure that each batch of ASP products meets the established active ingredient standards. Secondly, by formulating and following standardized protocols, it can be ensured that each batch of products can produce the same pharmacological effects. This involves not only the standardization of product formulations, but also clinical trials and bioequivalence studies to ensure the efficacy and safety of the product.

In addition, the use of biomarkers helps to monitor and evaluate the quality of products, ensuring the stability and consistency of their active ingredients. These biomarkers are screened by advanced biotechnology, which can reflect the metabolic process and bioavailability of ASP in human body. It is worth noting that improving the extraction method is also the key, which can involve the optimization of extraction conditions, such as the design of temperature, the selection of solvent type and the determination of extraction time, so as to maximize the purity and biological activity of angelica polysaccharide. Of course, this process also requires multidisciplinary cooperation, including the joint efforts of experts in the fields of chemistry, pharmacology and biotechnology.

Finally, following regulatory standards such as Good Manufacturing Practice (GMP) can ensure the standardization of the entire production process and the reliability of product quality. GMP standards not only require the production environment to be sterile and clean, but also require strict recording and monitoring of each step in the production process, including procurement, storage, processing, packaging of raw materials and quality inspection of final products. Through the implementation of these measures, we can provide consumers with stable quality and predictable effects of ASP derivative health products, so as to meet the growing market demand for high-quality natural health products. In the process of following GMP standards, special attention should also be paid to continuous improvement and innovation. Through the continuous introduction of advanced production equipment and technology, in order to improve production efficiency and product quality. At the same time, it is necessary to strengthen the training and management of employees to ensure that every production link meets the standards and can produce high-quality ASP products continuously and steadily.

On the other hand, with the continuous development of social economy, the public‘s attention to personal health is increasing day by day, which leads to an upward trend in the demand for natural medicines in the market, especially the large-scale production of medicinal materials such as ASP. However, in order to meet the needs of large-scale production, the research team recommended the use of synthetic simulation technology to prepare the active components of the natural drug ASP. Because this method can ensure the repeatability and effectiveness of the product, so as to meet the market demand. Synthetic mimetic active ingredients are widely recommended in large-scale production because they can ensure product consistency and reliability, thereby obtaining more repeatable and effective products. The structure and purity of active ingredients can be precisely controlled by chemical synthesis to ensure that each batch of products has the same efficacy and quality. This consistency is essential for pharmaceuticals, cosmetics and other products that require strict quality control. In addition, the production process of synthetic simulated active ingredients is often more controllable, which can meet the needs of large-scale industrial production, reduce the dependence on natural resources, reduce production costs, and have certain advantages in environmental protection. Therefore, the synthesis of simulated active ingredients can not only provide stable product quality, but also meet market demand and achieve the dual goals of economic benefits and environmental sustainability.

In recent years, with the rapid development and application of drug composition assays and computer-aided drug screening technologies, the pharmacological effects and targets of various Chinese medicinal components, such as polysaccharides and flavonoids, can be predicted and analyzed more comprehensively and systematically and compared with *in vitro* and *in vivo* data, which can help to systematically explain the pharmacological effects of ASP and its underlying mechanisms. This revealed the biological implications of the multipoint and synergistic effects of Chinese medicine.

## References

[B1] Basak T. Kanwar R. K. (2022). Iron imbalance in cancer: intersection of deficiency and overload. Cancer Med. 11 (20), 3837–3853. 10.1002/cam4.4761 35460205 PMC9582687

[B2] Bi S. J. Fu R. J. Li J. J. Chen Y. Y. Tang Y. P. (2021). The bioactivities and potential clinical values of *angelica sinensis* polysaccharides of *Angelica sinensis* polysaccharides. Nat. Prod. Commun. 16 (3). 10.1177/1934578X21997321

[B3] Cao P. Sun J. Sullivan M. A. Huang X. Wang H. Zhang Y. (2018). *Angelica sinensis* polysaccharide protects against acetaminophen-induced acute liver injury and cell death by suppressing oxidative stress and hepatic apoptosis *in vivo* and *in vitro* . Int. J. Biol. Macromol. 111, 1133–1139. 10.1016/j.ijbiomac.2018.01.139 29415408

[B4] Cao W. Li X. Hou Y. Fan H. Zhang X. Mei Q. (2008a). Structural analysis and antitumor effect of ASP APS-2a. Chin. Med. Mater. 31 (2), 261–266. 10.3321/j.issn:1001-4454.2008.02.031 18619275

[B5] Cao W. Li X. Q. Hou Y. Fan H. T. Zhang X. N. Mei Q. B. (2008b). Structural analysis and anti-tumor activity *in vivo* of polysaccharide APS-2a from *Angelica sinensis* . J. Chin. Med. Mater. 31 (2), 261–266. 10.3321/j.issn:1001-4454.2008.02.031 18619275

[B6] Cao W. Li X. Q. Liu L. Wang M. Fan H. T. Li C. (2006). Structural analysis of water-soluble glucans from the root of *Angelica sinensis* (Oliv.) Diels. Carbohydr. Res. 341 (11), 1870–1877. 10.1016/j.carres.2006.04.017 16682014

[B7] Cao W. Li X. Q. Wang X. Fan H. T. Zhang X. N. Hou Y. (2010a). A novel polysaccharide, isolated from *Angelica sinensis* (Oliv.) Diels induces the apoptosis of cervical cancer HeLa cells through an intrinsic apoptotic pathway. Phytomedicine 17 (8-9), 598–605. 10.1016/j.phymed.2009.12.014 20092988

[B8] Cao W. Li X. Q. Wang X. Li T. Chen X. Liu S. B. (2010b). Characterizations and anti-tumor activities of three acidic polysaccharides from *Angelica sinensis* (Oliv.) Diels. Int. J. Biol. Macromol. 46 (1), 115–122. 10.1016/j.ijbiomac.2009.11.005 19941888

[B9] Cao Y. (2019). Analysis of chemical constituents and pharmacological effects of *Angelica sinensis* . World 's latest Med. inf. Abstr. 19 (2), 93–95. 10.19613/j.cnki.1671-3141.2019.02.058

[B10] Chen J. (2010). On effects and mechanism of *Angelica sinensis* polysaccharides on glucose metabolism in experimental diabetic rats. J. Wuhan. Polytech. 9 (3), 93–95. 10.3969/j.issn.1671-931X.2010.03.023

[B11] Chen L. Huang G. (2018). The antiviral activity of polysaccharides and their derivatives. Int. J. Biol. Macromol. 115, 77–82. 10.1016/j.ijbiomac.2018.04.056 29654857

[B12] Chen R. Liu Y. Wang H. Xu H. Xu G. (2001a). Isolation and purification of ASP X-C-3-II and its composition. Chin. J. New Drugs. 10 (6), 431–432. 10.3321/j.issn:1003-3734.2001.06.010

[B13] Chen R. Wang H. Xu H. Xu G. Chang L. (2001b). Isolation, purification and identification of two polysaccharide fractions from Min *Angelica sinensis* . Chin. Med. Mater 24 (1), 36–37. 10.3321/j.issn:1001-4454.2001.01.016 11341028

[B14] Chen R. Xu G. Wang H. Xu H. Liu Y. (2001c). Isolation and structure of the polysaccharide XC-1 from *Angelica sinensis* . Chem. Bull. 3 (6), 372–374. 10.3969/j.issn.0441-3776.2001.06.009

[B15] Chen Y. Duan J. A. Qian D. Guo J. Song B. Yang M. (2010). Assessment and comparison of immunoregulatory activity of four hydrosoluble fractions of *Angelica sinensisin* vitro on the peritoneal macrophages in ICR mice. Int. Immunopharmacol. 10 (4), 422–430. 10.1016/j.intimp.2010.01.004 20093201

[B16] Chen Z. Cheng L. Zhang J. Cui X. (2022). Retraction Note: *Angelica sinensis* polysaccharide prevents mitochondrial apoptosis by regulating the Treg/Th17 ratio in aplastic anemia. BMC Complement. Med. Ther. 22 (1), 277. 10.1186/s12906-022-03752-5 36266649 PMC9585861

[B17] Cheng F. Zhang Y. Li Q. Zeng F. Wang K. (2020). Inhibition of dextran sodium sulfate-induced experimental colitis in mice by *angelica sinensis* polysaccharide. J. Med. Food. 23 (6), 584–592. 10.1089/jmf.2019.4607 32282259

[B18] Cheng Y. Zhou J. Li Q. Liu Y. Wang K. Zhang Y. (2016). The effects of polysaccharides from the root of *Angelica sinensis* on tumor growth and iron metabolism in H22-bearing mice. Food Funct. 7 (2), 1033–1039. 10.1039/c5fo00855g 26757699

[B19] Chinese Pharmacopoeia Commission. (2020). Pharmacopoeia of the people's Republic of China. Beijing, China: Ministry of Health of the People's Republic of China, 1 139.

[B20] Dienstmann R. Tabernero J. (2017). Cancer: a precision approach to tumour treatment. Nature 548 (7665), 40–41. 10.1038/nature23101 28723897

[B21] Ding X. Zhao X. Qiu Y. Chen K. Li Y. (2016). Effects of ASP on peripheral blood cells and immune function in cyclophosphamide-induced myelosuppressed mice. Health Prof. Educ. 34 (16), 153–155. Available at: https://d.wanfangdata.com.cn/Periodical/wszyjy201616084.

[B22] Erlin C. Xixiang L. Shanna W. Wang X. Niu M. Wang J. (2019). Study on the quality evaluation of *Angelica sinensis* based on the bioeffectiveness of blood activation. Chin. Med. Mater. 42 (4), 818–821. 10.13863/j.issn1001-4454.2019.04.024

[B23] Fu Z. Ma C. (2018). Effect of ASP on liver injury caused by cisplatin chemotherapy in H22 ascites tumor mice Pharmacology and clinical aspects of Chinese medicine. Pharmacol. Clin. Appl. Trad. Chin. Med. 34 (3), 68–72. 10.13412/j.cnki.zyyl.2018.03.017

[B24] Han D. Shinohara M. Ybanez M. D. Saberi B. Kaplowitz N. (2010). Signal transduction pathways involved in drug-induced liver injury. Handb. Exp. Pharmacol. 196, 267–310. 10.1007/978-3-642-00663-0_10 20020266

[B25] He X. Zhang Y. Wu H. Jiang R. (2012). Study on the protective effect of ASP on the hematopoietic system of radiation-injured mice. Chongqing Med. 41 (35), 3734–3736. 10.3969/j.issn.1671-8348.2012.35.019

[B26] Hong Y. Liu Y. Xiong X. Zhang Y. Wang H. (2002). Protective effects of ASP on the immune and hematopoietic functions of erythrocytes in radiation-injured mice. Med. Clin. Res. 19 (1), 31–32. 10.3969/j.issn.1671-7171.2002.06.014

[B27] Horvatits T. Drolz A. Trauner M. Fuhrmann V. (2019). Liver injury and failure in critical illness. Hepatology 70 (6), 2204–2215. 10.1002/hep.30824 31215660

[B28] Hou C. Yin M. Lan P. Wang H. Ji X. (2021). Recent progress in the research of *Angelica sinensis* (Oliv.) Diels polysaccharides: extraction, purification, structure and bioactivities. Chem. Biol. Technol. Agric. 8 (1), 13. 10.1186/s40538-021-00214-x

[B29] Hu Y. Kong X. Li X. Wang D. Liu J. Zhang B. (2004). Effect of 10 Chinese herbal ingredients on the proliferation and resistance of CEF to NDV infection. J. Anim. Husb. Vet. Med. 35 (3), 301–305. 10.3321/j.issn:0366-6964.2004.03.013

[B30] Hu Y. Liu J. Chen Y. Zhang B. Sun X. Wang X. (2003). Effect of Chinese medicinal ingredients on infected cells of infectious bursal virus. J. Anim. Husb. Vet. Med. 35 (12), 8–10. 10.3969/j.issn.0529-5130.2003.12.004

[B31] Huang X. (2010). Progress in the extraction methods of medicinal plant polysaccharides. Mod. Chem. Ind. 30 (12), 32–36. 10.16606/j.cnki.issn0253-4320.2010.12.007

[B32] Jia M. (2005). Study on the anti-AIDS effect of ASP sulfate and its mechanism. Master thesis. Xi’an: Fourth Military Medical University.

[B33] Jia M. Yang T. Yao X. Meng J. Meng J. Mei Q. (2007). Study on the antioxidant effect of ASP sulfate. Chin. Med. Mater. 30 (2), 185–188. 10.3321/j.issn:1001-4454.2007.02.026 17571770

[B34] Jia S. Wang D. Zhang X. Wang G. (2015). Effects of ASP on alcoholic hepatocyte injury in mice. Anat. Res. 37 (6), 468–471. Available at: https://d.wanfangdata.com.cn/periodical/jpxyj201506007. 10.20021/j.cnki.1671-0770.2015.06.007

[B35] Jiang J. Guo Y. J. Niu A. (2009). Extraction, characterization of *Angelica sinensis* polysaccharides and modulatory effect of the polysaccharides and Tai Chi exercise on oxidative injury in middle-aged women subjects. Carbohydr. Polym. 77 (2), 384–388. 10.1016/j.carbpol.2009.01.010

[B36] Jin M. Zhao K. Huang Q. Xu C. Shang P. (2012). Isolation, structure and bioactivities of the polysaccharides from *Angelica sinensis* (Oliv.) Diels: a review. Carbohydr. Polym. 89 (3), 713–722. 10.1016/j.carbpol.2012.04.049 24750855

[B37] Jin R. Li G. Ma S. (2007). Research on the preparation process of *Angelica* polysaccharide. Chin. Pat. Med. 29 (8), 1146–1150. 10.3969/j.issn.1001-1528.2007.08.016

[B38] Lee J. G. Hsieh W. T. Chen S. U. Chiang B. H. (2012). Hematopoietic and myeloprotective activities of an acidic *Angelica sinensis polysaccharide* on human CD34+ stem cells. J. Ethnopharmacol. 139 (3), 739–745. 10.1016/j.jep.2011.11.049 22155392

[B39] Li G. Xu L. (2017). Study on the effect of ASP on microRNA in the blood of radiation-injured mice. Northwest J. Pharmacol. 32 (2), 163–167. 10.3969/j.issn.1004-2407.2017.02.011

[B40] Li J. Liu X. Liu S. (2012). Process study of microwave-assisted extraction of *Angelica* polysaccharide. Anhui Agric. Sci. 40 (34), 16573–16574. 10.3969/j.issn.0517-6611.2012.34.025

[B41] Li M. M. Zhang Y. Wu J. Wang K. P. (2020). Polysaccharide from *angelica sinensis* suppresses inflammation and reverses anemia in complete freund's adjuvant-induced rats. Curr. Med. Sci. 40 (2), 265–274. 10.1007/s11596-020-2183-3 32337688

[B42] Li S. Wang X. Gui X. Dai L. (2005). Experimental study on the effect of ASP on the function of dendritic cells in hepatitis B virus transgenic mice. J. Pract. Diagn. Ther. 19 (5), 313–314. 10.3969/j.issn.1674-3474.2005.05.001

[B43] Liao F. Liu Y. Liu H. H. Hu J. Zhao S. Yang S. M. (2019). Effect of *Angelica sinensis* polysaccharide on the osteogenic differentiation of bone marrow mesenchymal stem cells of rats with high glucose levels. West China J. Stomatology 37 (2), 193–199. 10.7518/hxkq.2019.02.012 PMC7030159 31168987

[B44] Liu C. Li J. Meng F. Y. Liang S. X. Deng R. Li C. K. (2010). Polysaccharides from the root of *Angelica sinensis* promotes hematopoiesis and thrombopoiesis through the PI3K/AKT pathway. BMC Complement. Altern. Med. 10, 79. 10.1186/1472-6882-10-79 21176128 PMC3022894

[B45] Liu H. Zhang T. Wang M. Yu Y. Su L. Ji D. (2022a). Exploration of a new method for quality evaluation of *Angelica sinensis* based on *in vitro* blood tonic activity determination. J. Nanjing Univ. Chin. Med. 38 (12), 1110–1115. 10.14148/j.issn.1672-0482.2022.1110

[B46] Liu J. Y. Zhang Y. You R. X. Zeng F. Guo D. Wang K. P. (2012). Polysaccharide isolated from *Angelica sinensis* inhibits hepcidin expression in rats with iron deficiency anemia. J. Med. Food. 15 (10), 923–929. 10.1089/jmf.2012.2231 22985399 PMC3466919

[B47] Liu S. Yang Y. Qu Y. Guo X. Yang X. Cui X. (2020a). Structural characterization of a novel polysaccharide from Panax notoginseng residue and its immunomodulatory activity on bone marrow dendritic cells. Int. J. Biol. Macromol. 161, 797–809. 10.1016/j.ijbiomac.2020.06.117 32553971

[B48] Liu W. Li W. Sui Y. Li X. Q. Liu C. Jing H. (2019). Structure characterization and anti-leukemia activity of a novel polysaccharide from *Angelica sinensis* . Int. J. Biol. Macromol. 121, 161–172. 10.1016/j.ijbiomac.2018.09.213 30290264

[B49] Liu W. Xiao K. Ren L. Sui Y. Chen J. Zhang T. (2020b). Leukemia cells apoptosis by a newly discovered heterogeneous polysaccharide from *Angelica sinensis* . Carbohydr. Polym. 241, 116279. 10.1016/j.carbpol.2020.116279 32507223

[B50] Liu W. J. Li Z. Z. Feng C. X. Hu S. Yang X. Xiao K. (2022b). The structures of two polysaccharides from *Angelica sinensis* and their effects on hepatic insulin resistance through blocking RAGE. Carbohydr. Polym. 280, 119001. 10.1016/j.carbpol.2021.119001 35027136

[B51] Liu Y. Wei M. Song C. Li M. Yang M. Cao Z. (2020c). ASP inhibits apoptosis through upregulation of VEGF. Genom. Appl. Biol. 39 (3), 1307–1313. 10.13417/j.gab.039.001307

[B52] Liu Y. Yan C. An F. Wu Z. Su Y. Wang P. (2018). Effects of ASP on liver injury and immune damage in cadmium-contaminated rats. J. PLA Pharmacol. 34 (2), 101–104. 10.3969/j.issn.1008-9926.2018.02.001

[B53] Long Q. Maobao X. (2008). Comparative study on the quality of processed products from different habitats of *Angelica sinensis* . J. Gansu Coll. Traditional Chin. Med. 25 (2), 37–39. 10.3969/j.issn.1003-8450.2008.02.016

[B54] Lu Z. Wang L. Xu X. Zhang L. He J. Hua J. (2017a). Effects of *Angelica sinensis* and ASP on immune function impairment in SD rats caused by X-ray irradiation. Chin. J. Trad. Chin. Med. Inf. 24 (10), 44–48. 10.3969/j.issn.1005-5304.2017.10.011

[B55] Lu Z. Xu X. Wang L. (2017b). Protective effects of *Angelica sinensis* and ASP on radiation-induced liver and kidney injury in rats. J. PLA Med. 42 (9), 815. 10.11855/j.issn.0577-7402.2017.09.12

[B56] Luo Y. An F. Li N. Liu Y. Li C. Zhang Y. (2017). Effects of ASP and small molecule extracts on TGF-β1-induced expression of α-SMA and CTGF by HELF. Chin. Pharmacol. Clin. Pract. 33 (3), 73–78. 10.13412/j.cnki.zyyl.2017.03.022

[B57] Ma S. Li X. Lv Z. Wu J. (2022). Anti-inflammatory effects of ASP on rats with embolism during pregnancy and its effect on coagulation function. J. Guangzhou Univ. Chin. Med. 39 (3), 612–617. 10.13359/j.cnki.gzxbtcm.2022.03.025

[B58] Ma Y. Wu H. Jia M. Zhang Z. Wang J. Yue Z. (2024). Construction of iron oxide nanoparticles modified with *Angelica sinensis* polysac charide for the treatment of iron deficiency anemia. J. Nanoparticle Res. 26 (11), 250–250. 10.1007/s11051-024-06169-y

[B59] Nai J. Zhang C. Shao H. Li B. Li H. Gao L. (2021). Extraction, structure, pharmacological activities and drug carrier applications of *Angelica sinensis* polysaccharide. Int. J. Biol. Macromol. 183, 2337–2353. 10.1016/j.ijbiomac.2021.05.213 34090852

[B60] Niu X. Zhang J. Ling C. Bai M. Peng Y. Sun S. (2018). Polysaccharide from *Angelica sinensis* protects H9c2 cells against oxidative injury and endoplasmic reticulum stress by activating the ATF6 pathway. J. Int. Med. Res. 46 (5), 1717–1733. 10.1177/0300060518758863 29517941 PMC5991254

[B61] Ouyang X. He Y. Zhu J. He J. Ma X. Ding Y. (2005). Comprehensive quality evaluation of Gansu *Angelicae Sinensis* Radix with different commodity specifications. J. Traditional Chin. Med. 33 (4), 12–14. 10.19664/j.cnki.1002-2392.2005.04.007

[B62] Pan Q. Li Y. Liu D. Zhao H. (2015). Regulatory effects of ASP on the level of T lymphocyte subsets in peyer, spatches junction in mice with colitis. Jiangxi Trad. Chin. Med. 46 (10), 33–37. Available at: https://d.wanfangdata.com.cn/Periodical/jxzyy201510015.

[B63] Pan S. Jiang L. Wu S. (2018). Stimulating effects of polysaccharide from *Angelica sinensis* on the nonspecific immunity of white shrimps (*Litopenaeus* vannamei). Fish. Shellfish Immunol. 74, 170–174. 10.1016/j.fsi.2017.12.067 29305988

[B64] Pantopoulos K. Porwal S. K. Tartakoff A. Devireddy L. (2012). Mechanisms of mammalian iron homeostasis. Biochemistry 51 (29), 5705–5724. 10.1021/bi300752r 22703180 PMC3572738

[B65] Pu X. Ma X. Liu L. Ren J. Li H. Li X. (2016). Structural characterization and antioxidant activity *in vitro* of polysaccharides from *Angelica* and *Astragalus* . Carbohydr. Polym. 137, 154–164. 10.1016/j.carbpol.2015.10.053 26686116

[B66] Qian W. Cai X. Qian Q. Wang D. Zhang L. (2020). *Angelica sinensis* polysaccharide suppresses epithelial-mesenchymal transition and pulmonary fibrosis via a DANCR/AUF-1/FOXO3 regulatory Axis. Aging Dis. 11 (1), 17–30. 10.14336/ad.2019.0512 32010478 PMC6961774

[B67] Ren F. Li J. Wang Y. Wang Y. Feng S. Yuan Z. (2018). The effects of *angelica sinensis* polysaccharide on tumor growth and iron metabolism by regulating hepcidin in tumor-bearing mice. Cell Physiol. biochem. 47 (3), 1084–1094. 10.1159/000490185 29843136

[B68] Shang P. Qian A. R. Yang T. H. Jia M. Mei Q. B. Cho C. H. (2003). Experimental study of anti-tumor effects of polysaccharides from *Angelica sinensis* . World J. Gastroenterol. 9 (9), 1963–1967. 10.3748/wjg.v9.i9.1963 12970885 PMC4656653

[B69] Song P. Jianbang Z. Zhang J. Ding Y. (2008). Quality investigation of different cultivation methods and different specifications of *Angelica sinensis* . 21(9), 49–50. 10.3969/j.issn.10046852.2008.09.035

[B70] Song X. Kong J. Song J. Pan R. Wang L. (2021). *Angelica sinensis* polysaccharide alleviates myocardial fibrosis and oxidative stress in the heart of hypertensive rats. Comput. Math. Methods Med. 2021, 6710006. 10.1155/2021/6710006 34527077 PMC8437656

[B71] Sui Y. Liu W. Tian W. Li X. Q. Cao W. (2019). A branched arabinoglucan from *Angelica sinensis* ameliorates diabetic renal damage in rats. Phytother. Res. 33 (3), 818–831. 10.1002/ptr.6275 30672023

[B72] Sun Y. Cui S. W. Tang J. Gu X. (2010). Structural features of pectic polysaccharide from *Angelica sinensis* (Oliv.) Diels. Carbohydr. Polym. 80 (2), 544–550. 10.1016/j.carbpol.2009.12.030

[B73] Sun Y. Gu X. Tang J. Li D. (2006). Isolation, purification and preliminary structural analysis of water-soluble polysaccharides from *Angelica sinensis* . J. Food Biotechnol. 25 (1), 1–4. 10.3321/j.issn:1673-1689.2006.01.001

[B74] Sun Y. Ma G. Tang J. (2009). Study on the protective effect of ASP on subchronic radiation injury in mice. Chin. J. Food. 9 (4), 33–37. 10.3969/j.issn.1009-7848.2009.04.006

[B75] Sun Y. Tang J. Gu X. Li D. (2005). Water-soluble polysaccharides from *Angelica sinensis*: preparation, characterization and bioactivity. Int. J. Biol. Macromol. 36 (5), 283–289. 10.1016/j.ijbiomac.2005.07.005 16129482

[B76] Tang Z. Long Q. Liu X. Liao W. Zhang X. Wang S. (2020). ASP inhibits the growth, migration and invasion of cervical cancer Hela cells by regulating the p38 pathway. Chin. J. Immunol. 36 (3), 332–337. 10.3969/j.issn.1000-484X.2020.03.014

[B77] Tian S. Hao C. Xu G. Yang J. Sun R. (2017). Optimization conditions for extracting polysaccharide from *Angelica sinensis* and its antioxidant activities. J. Food Drug Anal. 25 (4), 766–775. 10.1016/j.jfda.2016.08.012 28987352 PMC9328866

[B78] Tian Y. Shen X. Hu T. Liang Z. Ding Y. Dai H. (2024). Structural analysis and blood-enriching effects comparison based on biological potency of *Angelica sinensis* polysaccharides. Front. Pharmacol. 15, 1405342. 10.3389/fphar.2024.1405342 38953103 PMC11215113

[B79] Wang D. Zhang H. Wang G. Jia S. (2016a). Morphological and structural observation of the effect of ASP on alcoholic liver injury in mice. J. Chang. Med. Coll. 30 (4), 256–258. 10.3969/j.issn.1006-0588.2016.04.005

[B80] Wang G. Jing P. Jia S. (2017a). Effect of ASP on the level of intercellular adhesion molecule 1 on the surface of bone marrow hematopoietic stem cells and stromal cells in mice. Chin. Tissue Eng. Res. 21 (21), 3293–3298. 10.3969/j.issn.2095-4344.2017.21.003

[B81] Wang J. Hu Y. Zhang F. Wang D. Zhao X. Zhang J. (2011a). Effect of eight sulfated polysaccharides on the ability of Newcastle disease virus to infect chicken embryo fibroblasts. J. Nanjing Agric. Univ. 34 (1), 118–122. 10.7685/j.issn.1000-2030.2011.01.022

[B82] Wang K. Cao P. Shui W. Yang Q. Tang Z. Zhang Y. (2015a). *Angelica sinensis* polysaccharide regulates glucose and lipid metabolism disorder in prediabetic and streptozotocin-induced diabetic mice through the elevation of glycogen levels and reduction of inflammatory factors. Food Funct. 6 (3), 902–909. 10.1039/c4fo00859f 25630053

[B83] Wang K. Cao P. Wang H. Tang Z. Wang N. Wang J. (2016b). Chronic administration of *Angelica sinensis* polysaccharide effectively improves fatty liver and glucose homeostasis in high-fat diet-fed mice. Sci. Rep. 6, 26229. 10.1038/srep26229 27189109 PMC4870572

[B84] Wang K. Chen Z. Zhang Y. Wang J. Hu M. Dai L. (2007a). Experimental study of *Angelica sinensis* polysacch aride-iron complex on rats with iron deficiency anemia. Chin. J. New Drugs Clin. Rem. 26 (6), 413–416. 10.3969/j.issn.1007-7669.2007.06.004

[B85] Wang K. Song Z. Wang H. Li Q. Cui Z. Zhang Y. (2016c). *Angelica sinensis* polysaccharide attenuates concanavalin A-induced liver injury in mice. Int. Immunopharmacol. 31, 140–148. 10.1016/j.intimp.2015.12.021 26741264

[B86] Wang K. Tang Z. Zheng Z. Cao P. Shui W. Li Q. (2016d). Protective effects of *Angelica sinensis* polysaccharide against hyperglycemia and liver injury in multiple low-dose streptozotocin-induced type 2 diabetic BALB/c mice. Food Funct. 7 (12), 4889–4897. 10.1039/c6fo01196a 27813540

[B87] Wang K. Wang J. Song M. Wang H. Xia N. Zhang Y. (2020). *Angelica sinensis* polysaccharide attenuates CCl(4)-induced liver fibrosis via the IL-22/STAT3 pathway. Int. J. Biol. Macromol. 162, 273–283. 10.1016/j.ijbiomac.2020.06.166 32569681

[B88] Wang K. Wu J. Cheng F. Huang X. Zeng F. Zhang Y. (2017b). Acidic polysaccharide from *Angelica sinensis* reverses anemia of chronic disease involving the suppression of inflammatory hepcidin and NF-κB activation. Oxid. Med. Cell Longev. 2017, 7601592. 10.1155/2017/7601592 29147463 PMC5632906

[B89] Wang K. Wu J. Xu J. Gu S. Li Q. Cao P. (2018). Correction of anemia in chronic kidney disease with *Angelica sinensis* polysaccharide via restoring EPO production and improving iron availability. Front. Pharmacol. 9, 803. 10.3389/fphar.2018.00803 30108502 PMC6079227

[B90] Wang K. P. Zeng F. Liu J. Y. Guo D. Zhang Y. (2011b). Inhibitory effect of polysaccharides isolated from *Angelica sinensis* on hepcidin expression. J. Ethnopharmacol. 134 (3), 944–948. 10.1016/j.jep.2011.02.015 21333724

[B91] Wang L. Jiang R. Song S. D. Hua Z. S. Wang J. W. Wang Y. P. (2015b). *Angelica sinensis* polysaccharide induces erythroid differentiation of human chronic myelogenous leukemia k562 cells. Asian pac. J. Cancer Prev. 16 (9), 3715–3721. 10.7314/apjcp.2015.16.9.3715 25987027

[B92] Wang L. Xu X. Lu Z. (2017c). Study on the protection of ASP against liver damage by X-ray radiation in rats. Chin. Herb. Med. 48 (20), 4284.

[B93] Wang P. P. Zhang Y. Dai L. Q. Wang K. P. (2007b). Effect of *Angelica sinensis* polysaccharide-iron complex on iron deficiency anemia in rats. Chin. J. Integr. Med. 13 (4), 297–300. 10.1007/s11655-007-0297-0 18180896

[B94] Wang S. Shi S. Cui J. Ding L. Wang Z. Hu Z. (2007c). Isolation and purification of a heteropolysaccharide from *Angelica sinensis* . Chin. J. Pharm. Sci. 42 (16), 1255–1258. 10.3321/j.issn:1001-2494.2007.16.016

[B95] Wang Y. Li X. Zhao P. Qu Z. Bai D. Gao X. (2019). Physicochemical characterizations of polysaccharides from *Angelica Sinensis* Radix under different drying methods for various applications. Int. J. Biol. Macromol. 121, 381–389. 10.1016/j.ijbiomac.2018.10.035 30315881

[B96] Wang Y. Wang X. Zhang X. Lin X. Wang X. An Y. (2010). Effects of ASP on lung function and lung coefficient in rats with pulmonary fibrosis. Gansu Trad. Chin. Med. 23 (11), 28–31. 10.3969/j.issn.1004-6852.2010.11.013

[B98] Wu G. Yang X. Yi D. Chen H. Yang Y. Yang Z. (2018a). Quality evaluation of *Angelica sinensis* and its different medicinal parts based on grey correlation degree. Chin. J. Traditional Chin. Med. Inf. 25(5), 77–81.

[B99] Wu G. Yang X. Yi D. Chen H. Yang Y. Yang Z. (2018b). Fingerprint and component analysis of different medicinal parts of *Angelica sinensis* . Chin. Tradit. Plant Med. 40(04), 890–894. 10.3969/j.issn.1001-1528.2018.04.025

[B100] Wu J. Meng J. Zhang Y. Wu H. Liu M. Wei N. (2017). Effects of ASP iron complex on blood routine and immune and antioxidant functions in rats with iron deficiency anemia model. J. Gansu Univ. Chin. Med. 34 (1). 10.16841/j.issn1003-8450.2017.01.03

[B101] Wu J. Shao J. Wu J. Li H. Li W. Zhang Y. (2015). Optimization of the extraction process of ASP based on orthogonal design. J. Gansu Coll. Trad. Chin. Med. 32 (5), 22–25. Available at: https://kns.cnki.net/kcms2/article/abstract?v=IILC1c-FiAExHdArDj3r2ZSOAi4a9IqfZMl2Sej1sQoNhYZYCGPkYUIEjMHjMJqy9EdjLVdxSX6660ciYKvcgytyhNYhE3DwUxCtn7h20aG4P2atlaaCwB1mfxrOfLLA1ZWvKS2_-fsgljSWlsSeVw==&uniplatform=NZKPT&language=CHS.

[B102] Wu S. Li J. Chen S. Zhu X. (2012). Experimental study on the antitumor effects of sulfated ASP. Shi-Zhen Guomao. 23 (2), 319–320. 10.3969/j.issn.1008-0805.2012.02.029

[B103] Wu S. Li J. Chen S. Zhu X. (2013). Preparation and antitumor activity of sulfated ASP. Chin. J. Hosp. Pharm. 33 (22), 1832–1835. 10.13286/j.cnki.chinhosppharmacyj.2013.22.003

[B104] Xu C. Ni S. Zhuang C. Li C. Zhao G. Jiang S. (2021). Polysaccharide from *Angelica sinensis* attenuates SNP-induced apoptosis in osteoarthritis chondrocytes by inducing autophagy via the ERK1/2 pathway. Arthritis Res. Ther. 23 (1), 47. 10.1186/s13075-020-02409-3 33514407 PMC7847159

[B105] Xu C. Shen L. Yuan W. (2022). ASP inhibits oxidative stress injury and inflammatory response in osteoarthritic chondrocytes via Wnt/β-catenin signaling pathway. Shaanxi TCM 43 (6), 700–703. 10.3969/j.issn.1000-7369.2022.06.005

[B106] Xu X. Lu Z. Wang L. Zhang L. An F. He J. (2017). Study on the damage of bone marrow and spleen in rats by ASP protection against X-rays. Chin. J. Pharmacol. 33 (11), 1553–1558. 10.3969/j.issn.1001-1978.2017.11.015

[B107] Xu R. Xu J. Li Y. C. Dai Y. T. Zhang S. P. Wang G. (2020). Integrated chemical and transcriptomic analyses unveils synthetic characteristics of different medicinal root parts of *Angelica sinensis* . Chin. Herb. Med. 12 (1), 9–28. 10.1016/j.chmed.2019.07.003 PMC9476730 36117566

[B108] Yang J. Shao X. Jiang J. Sun Y. Wang L. Sun L. (2018). *Angelica sinensis* polysaccharide inhibits proliferation, migration, and invasion by downregulating microRNA-675 in human neuroblastoma cell line SH-SY5Y. Cell Biol. Int. 42 (7), 867–876. 10.1002/cbin.10954 29465760

[B109] Yang T. Jia M. Meng J. Wu H. Mei Q. (2006). Immunomodulatory activity of polysaccharide isolated from *Angelica sinensis* . Int. J. Biol. Macromol. 39 (4-5), 179–184. 10.1016/j.ijbiomac.2006.02.013 16839602

[B110] Yang T. Jia M. Zhou S. Pan F. Mei Q. (2012). Antivirus and immune enhancement activities of sulfated polysaccharide from *Angelica sinensis* . Int. J. Biol. Macromol. 50 (3), 768–772. 10.1016/j.ijbiomac.2011.11.027 22155400

[B111] Yang X. Zhao Y. Li G. Wang Z. Lv Y. (2008a). Chemical composition and immuno-stimulating properties of polysaccharide biological response modifier isolated from Radix *Angelica sinensis* . Food Chem. 106 (1), 269–276. 10.1016/j.foodchem.2007.05.085

[B112] Yang X. Zhao Y. Lv Y. (2008b). *In vivo* macrophage activation and physicochemical property of the different polysaccharide fractions purified from *Angelica sinensis* . Carbohydr. Polym. 71 (3), 372–379. 10.1016/j.carbpol.2007.06.002

[B113] Yu F. Li H. Meng Y. Yang D. (2013). Extraction optimization of *Angelica sinensis* polysaccharides and its antioxidant activity *in vivo* . Carbohydr. Polym. 94 (1), 114–119. 10.1016/j.carbpol.2013.01.050 23544518

[B114] Zhang J. Yin D. D. Luo G. (1998). Extraction of ASP and preparation of punch. Chin. J. Hosp. Pharm. 18 (5), 220–221. 10.3321/j.issn:1001-5213.1998.05.017

[B115] Zhang L. Huang R. (1999). Purification, identification and structural study of water-soluble polysaccharide fractions As-IIIa and As-IIIb from *Angelica sinensis* . J. Laser Biol. 8 (2), 46–49. 10.3969/j.issn.1007-7146.1999.02.010

[B116] Zhang L. Lu Z. Xu X. Wang L. Yong W. Li J. (2017). Study on the protective effect of ASP on oxidative stress in the kidney of radiation rats and Nrf2-related mechanism. Chin. Pharmacol. Clin. Pract. 33 (5), 63–66. 10.13412/j.cnki.zyyl.2017.05.017

[B117] Zhang L. Lu Z. Xu X. Wei K. Li Y. Liu Y. (2020). Study on the protective effect of ASP on radiation-induced intestinal barrier damage in SD rats. Shizhen Guomao Guomao 31 (12), 2847–2850. 10.3969/j.issn.1008-0805.2020.12.008

[B118] Zhang M. Wang X. (2018). Advances in the pathogenesis and diagnosis of inflammatory anemia associated with iron homeostasis dysregulation. Diagn. Theor. Pract. 17 (5), 601. 10.16150/j.1671-2870.2018.05.023

[B119] Zhang P. Wang Q. Chen H. Li X. Dou J. Chen J. (2009). *In vitro* inhibition of apotosis in HCMV-infected megakaryocytes by ASP. Chin. J. Exp. Hematol. 17 (1), 193–197. Available at: https://d.wanfangdata.com.cn/Periodical/zgsyxyxzz200901042.

[B120] Zhang Q. Yu H. Wang Q. Sun X. Xing K. Guo Y. (2016a). Extraction, isolation, and purification of Angelica polysaccharide. Hebei Fish. (3), 1–3. 10.3969/j.issn.1004-6755.2016.03.001

[B121] Zhang S. B. Lv J. X. (2013). Comparison on four extraction methods of polysaccharide from *Angelica sinensis* Tianjin Agric. Sci. Res. 19 (10), 33–36. 10.3969/j.issn.1006-6500.2013.10.009

[B122] Zhang X. Wang Q. Li C. Zhang Y. Li K. Hu S. (2012a). New process of enzymatic extraction of *Angelica* polysaccharides. J. Trad. Chin. Med. 40 (3), 96–100. 10.3969/j.issn.1002-2392.2012.03.033

[B124] Zhang Y. Cheng Y. Wang N. Zhang Q. Wang K. (2014). The action of JAK, SMAD and ERK signal pathways on hepcidin suppression by polysaccharides from *Angelica sinensis* in rats with iron deficiency anemia. Food Funct. 5 (7), 1381–1388. 10.1039/c4fo00006d 24752529

[B125] Zhang Y. He Z. Liu X. Chen Z. Sun J. Wu Z. (2019). Oral administration of *Angelica sinensis* polysaccharide protects against pancreatic islets failure in type 2 diabetic mice: pancreatic β-cell apoptosis inhibition. J. Funct. Foods. 54, 361–370. 10.1016/j.jff.2019.01.037

[B126] Zhang Y. Li M. M. Zeng F. Yao C. Wang K. P. (2012b). Study to establish the role of JAK2 and SMAD1/5/8 pathways in the inhibition of hepcidin by polysaccharides from *Angelica sinensis* . J. Ethnopharmacol. 144 (2), 433–440. 10.1016/j.jep.2012.09.040 23036813

[B127] Zhang Y. Zhou T. Wang H. Cui Z. Cheng F. Wang K. P. (2016b). Structural characterization and *in vitro* antitumor activity of an acidic polysaccharide from *Angelica sinensis* (Oliv.) Diels. Carbohydr. Polym. 147, 401–408. 10.1016/j.carbpol.2016.04.002 27178946

[B128] Zhang Z. (2006). Application of microwave-assisted extraction technology in polysaccharide research. Chin. Herb. Med. (4), 630–632. 10.7501/j.issn.0253-2670.2006.4.260

[B129] Zhao Y. Shi Y. Yang H. Mao L. (2016). Extraction of *Angelica sinensis* polysaccharides using ultrasound-assisted way and its bioactivity. Int. J. Biol. Macromol. 88, 44–50. 10.1016/j.ijbiomac.2016.01.113 26845475

[B130] Zhou S. Zhang B. Liu X. Teng Z. Huan M. Yang T. (2009). A new natural angelica polysaccharide based colon-specific drug delivery system. J. Pharm. Sci. 98 (2), 4756–4768. 10.1002/jps.21790 19408300

[B131] Zhou T. (2018). Isolation analysis, structure identification and biological activity of ASP. Wuhan: Huazhong University of Science and Technology.

[B132] Zhou W. J. Wang S. Hu Z. Zhou Z. Y. Song C. J. (2015). *Angelica sinensis* polysaccharides promotes apoptosis in human breast cancer cells via CREB-regulated caspase-3 activation. Biochem. Biophys. Res. Commun. 467 (3), 562–569. 10.1016/j.bbrc.2015.09.145 26431878

[B133] Zhou X. Wang Q. Zhu X. Pan R. Pan S. Zhu F. (2018). Effects of ASP on TLR4/MyD88/NF-κB pathway inhibition in DPN rats. Chin. Clin. Pharmacol. Ther. 23 (12), 1340–1347. 10.12092/j.issn.1009-2501.2018.12.004

[B134] Zhou Y. Guo X. Chen W. Liu J. (2019). *Angelica* polysaccharide mitigates lipopolysaccharide-evoked inflammatory injury by regulating microRNA-10a in neuronal cell line HT22. Artif. Cells Nanomed. Biotechnol. 47 (1), 3194–3201. 10.1080/21691401.2019.1614595 31353963

[B135] Zhuang C. Xu N. W. Gao G. M. Ni S. Miao K. S. Li C. K. (2016). Polysaccharide from *Angelica sinensis* protects chondrocytes from H2O2-induced apoptosis through its antioxidant effects *in vitro* . Int. J. Biol. Macromol. 87, 322–328. 10.1016/j.ijbiomac.2016.02.031 26893055

[B136] Zou Y. F. Fu L. Cai W. Jiang Q. X. Peng X. Li L. X. (2022). The comparison of preliminary structure and intestinal anti-inflammatory and anti-oxidative activities of polysaccharides from different root parts of *Angelica sinensis (Oliv.) Diels* . J. Ethnopharmacol. 295, 115446, 10.1016/j.jep.2022.115446 35675860

